# Maize endonuclease NOB1 couples ribosome biogenesis with translational regulation during kernel development

**DOI:** 10.1093/plcell/koag330

**Published:** 2026-10-28

**Authors:** Haixiang Ruan, Shun Peng, Yiqing You, Xin Ye, Xiaolong Zhao, Lu Ding, Binbin Zhao, Jun Yang

**Affiliations:** National Engineering Laboratory of Crop Stress Resistance Breeding, Anhui Agricultural University, No. 130 Changjiang West Road, Anhui Province, Hefei 230036, China; National Engineering Laboratory of Crop Stress Resistance Breeding, Anhui Agricultural University, No. 130 Changjiang West Road, Anhui Province, Hefei 230036, China; National Engineering Laboratory of Crop Stress Resistance Breeding, Anhui Agricultural University, No. 130 Changjiang West Road, Anhui Province, Hefei 230036, China; National Engineering Laboratory of Crop Stress Resistance Breeding, Anhui Agricultural University, No. 130 Changjiang West Road, Anhui Province, Hefei 230036, China; National Engineering Laboratory of Crop Stress Resistance Breeding, Anhui Agricultural University, No. 130 Changjiang West Road, Anhui Province, Hefei 230036, China; National Engineering Laboratory of Crop Stress Resistance Breeding, Anhui Agricultural University, No. 130 Changjiang West Road, Anhui Province, Hefei 230036, China; National Engineering Laboratory of Crop Stress Resistance Breeding, Anhui Agricultural University, No. 130 Changjiang West Road, Anhui Province, Hefei 230036, China; National Engineering Laboratory of Crop Stress Resistance Breeding, Anhui Agricultural University, No. 130 Changjiang West Road, Anhui Province, Hefei 230036, China

## Abstract

NOB1-mediated cleavage of 20S pre-rRNA represents a critical quality-control step during cytoplasmic maturation of the eukaryotic 40S ribosomal subunit, yet its developmental significance in crops remains largely unexplored. Here, we identified 2 highly conserved maize NOB1 paralogs, ZmNOB1a and ZmNOB1b, both exhibiting RNA-binding and endonuclease activities and associating with pre-40S ribosomal particles. Genetic analyses demonstrated that the 2 paralogs function redundantly, but with unequal contributions, to support gametophyte viability and kernel development. Simultaneous disruption of ZmNOB1 activity resulted in severely impaired male gametophyte transmission, markedly reduced female gamete viability, and profound defects in embryo and endosperm development. Molecular analyses further revealed widespread disruption of pre-rRNA maturation, including defective D-site cleavage, accompanied by impaired ribosome assembly and reduced translational capacity. Consistent with these molecular defects, mutant kernels exhibited decreased starch and storage protein accumulation, together with elevated levels of free ribosomal subunits and reduced 80S monosomes and polysomes. Moreover, ZmNOB1 exhibited co-sedimentation and directly interacted with the translation initiation factor eIF4A, implying a potential functional coordination between late ribosome maturation and translation initiation. Collectively, our findings establish ZmNOB1-mediated pre-rRNA processing as a key determinant of translational competence, reproductive fitness, and seed filling in maize.

## Introduction

Ribosome biogenesis is a fundamental and highly coordinated process that underpins protein synthesis, cellular growth, and development in all eukaryotes. The eukaryotic 80S ribosome consists of a 60S large subunit (LSU) and a 40S small subunit (SSU) (Sáez-Vásquez and Delseny 2019). In plants, the LSU contains the 25S, 5.8S, and 5S rRNAs together with more than 40 ribosomal proteins (RPs), whereas the SSU comprises the 18S rRNA and over 30 RPs (Weis et al. 2015). Ribosome biogenesis initiates in the nucleolus with RNA polymerase I-mediated transcription of the polycistronic 45S pre-rRNA, which contains the 18S, 5.8S, and 25S rRNA sequences separated by internal transcribed spacers (ITS1 and ITS2) and flanked by external transcribed spacers (5′-ETS and 3′-ETS) (Boisvert et al. 2007). These spacer sequences are subsequently removed through a series of highly coordinated endonucleolytic and exonucleolytic processing events to generate mature rRNAs (Fernández-Pevida et al. 2015; Tomecki et al. 2017). By contrast, 5S rRNA is independently transcribed by RNA polymerase III and incorporated into the LSU during ribosome assembly (Ciganda and Williams 2011; Tomecki et al. 2017).

Although the overall framework of pre-rRNA processing is conserved across eukaryotes, plants possess several distinct maturation features. In yeast, the major 5′-ETS-first pathway initiates with cleavage at sites A0 and A1 within the 5′-ETS, followed by cleavage at site A2, which separates precursors destined for SSU and LSU maturation (Kos and Tollervey 2010; Henras et al. 2015). Plants additionally employ an ITS1-first pathway initiated by cleavage at site A3, producing the P-A3 and 27S-A3 intermediates (Hang et al. 2018; Sáez-Vásquez and Delseny 2019; Liu et al. 2020). Subsequent trimming and processing steps generate the 18S-A2 precursor, which is exported to the cytoplasm for terminal maturation. At this stage, the 18S precursor still retains a short 3′ extension corresponding to the D-A2 fragment. Removal of this fragment by D-site cleavage constitutes the final maturation step of the 40S subunit and functions as a critical quality-control checkpoint that prevents immature ribosomes from prematurely entering translation (Woolford and Baserga 2013; Ameismeier et al. 2018).

Ribosome assembly is exceptionally complex and requires more than 250 ribosome biogenesis factors (RBFs) that coordinate rRNA processing, folding, modification, and RP incorporation (Woolford and Baserga 2013). Consistent with the central role of ribosome biogenesis in cellular homeostasis, mutations affecting RBFs in plants frequently cause severe developmental defects, including embryo lethality, defective gametophyte development, and impaired seed formation (Shi et al. 2005; Lange et al. 2011; Wang et al. 2016; Shanmugam et al. 2017). In maize, several RBFs essential for rRNA processing and kernel development have been characterized. Urb2 is required for efficient processing of 35S/33S and 18S-related precursors during endosperm development (Wang et al. 2018). *Shrek1* encodes a WD40-repeat protein involved in cleavage at site A3, and its mutation causes embryo lethality and defective endosperm differentiation (Liu et al. 2022). Rcl1 participates in cleavages at sites P′, A1, and A2, whereas Dek51 and Dek58 regulate multiple pre-rRNA maturation steps associated with 18S and 25S biogenesis (Wang et al. 2022a, 2024; Ma et al. 2024). Notably, these characterized factors function predominantly in the nucleus, whereas the mechanisms governing terminal cytoplasmic maturation of the 40S subunit in crops remain poorly understood.

NOB1 is an evolutionarily conserved PilT N-terminus (PIN)-domain endonuclease that catalyzes D-site cleavage during 18S rRNA maturation in archaea, yeast, animals, and Arabidopsis (Fatica et al. 2003; Veith et al. 2012; Missbach et al. 2013). The protein contains a catalytic PIN domain responsible for RNA cleavage and a Zn-ribbon domain that mediates association with pre-40S particles (Lamanna and Karbstein 2009). Structural studies in human cells further revealed that NOB1 activity is tightly controlled during late cytoplasmic maturation. In immature pre-40S particles, the assembly factor PNO1 masks the 18S rRNA 3′ end and maintains NOB1 in an inactive state. Subsequent remodeling mediated by the kinase RIOK1 displaces PNO1 and activates NOB1 cleavage activity, thereby licensing mature 40S subunits for translation initiation (Ameismeier et al. 2018, 2020). Despite its conserved role as a key checkpoint factor during ribosome maturation, the developmental functions and regulatory mechanisms of NOB1 in crops remain largely unexplored.

Translation initiation in eukaryotes begins with assembly of the 43S pre-initiation complex, consisting of the mature 40S subunit, eIF1, eIF1A, eIF3, eIF5, and the eIF2·GTP·Met-tRNA_i_^Met^ ternary complex (Brito Querido et al. 2024a). Recruitment of the 43S complex to capped mRNA depends on the eIF4F complex, composed of eIF4E, eIF4G, and the DEAD-box RNA helicase eIF4A, which recognizes the mRNA 5′ cap and unwinds secondary structures during ribosome scanning (Wang et al. 2022b). Recent proximity-labeling studies in human cells identified associations between NOB1/PNO1 and several translation initiation factors, including eIF4B and eIF4G2 (Xu et al. 2025), raising the possibility that late-stage ribosome maturation is functionally coordinated with translation initiation. Whether such coupling occurs in plants, however, remains unknown.

Here, we identified 2 maize *NOB1* paralogs, *ZmNOB1a* and *ZmNOB1b*, and demonstrated that they function redundantly yet unequally in gametophyte transmission, kernel development, and ribosome biogenesis. Simultaneous disruption of *ZmNOB1* function causes defective D-site cleavage, widespread abnormalities in pre-rRNA processing, impaired ribosome assembly, and reduced global translation. Furthermore, ZmNOB1 co-sedimented with eIF4A and physically associates with the eIF4F complex, suggesting a potential mechanistic link between late ribosome maturation and translation initiation. Together, our findings establish ZmNOB1 as a central regulator coupling ribosome biogenesis to translational capacity during maize kernel development and provide new insights into translational regulation in cereal crops.

## Results

### Maize NOB1 paralogs encode evolutionarily conserved RNA-binding endonuclease

NOB1 functions as the endonuclease responsible for D-site cleavage during 18S rRNA maturation in archaea, yeast, and animals, underscoring its deeply conserved role in ribosome biogenesis (Fatica et al. 2003; Veith et al. 2012; Cerezo et al. 2019). Two maize paralogs, designated ZmNOB1a (Zm00001d014988) and ZmNOB1b (Zm00001d054047), were identified in the B73 reference genome. ZmNOB1a and ZmNOB1b share 89.61% amino acid sequence similarity (Figure S1a; Data Set S1). AlphaFold-based structural prediction revealed nearly superimposable architectures for the 2 proteins (Fig. 1a), each comprising a conserved catalytic PIN domain and a C-terminal Zn-ribbon domain. Sequence divergence among NOB1 orthologs is largely confined to the N-terminal region and the linker between the 2 domains (Figure S1, a and b). Phylogenetic analysis further demonstrated broad conservation of NOB1 proteins across eukaryotic lineages, although duplicated paralogs were retained in multiple angiosperm species (Figure S1c; Data Set S2). Consistent with this evolutionary conservation, structural comparison revealed a highly preserved PIN-domain configuration among maize, Arabidopsis, and yeast NOB1 proteins (Figure S1d).

**Figure 1:**
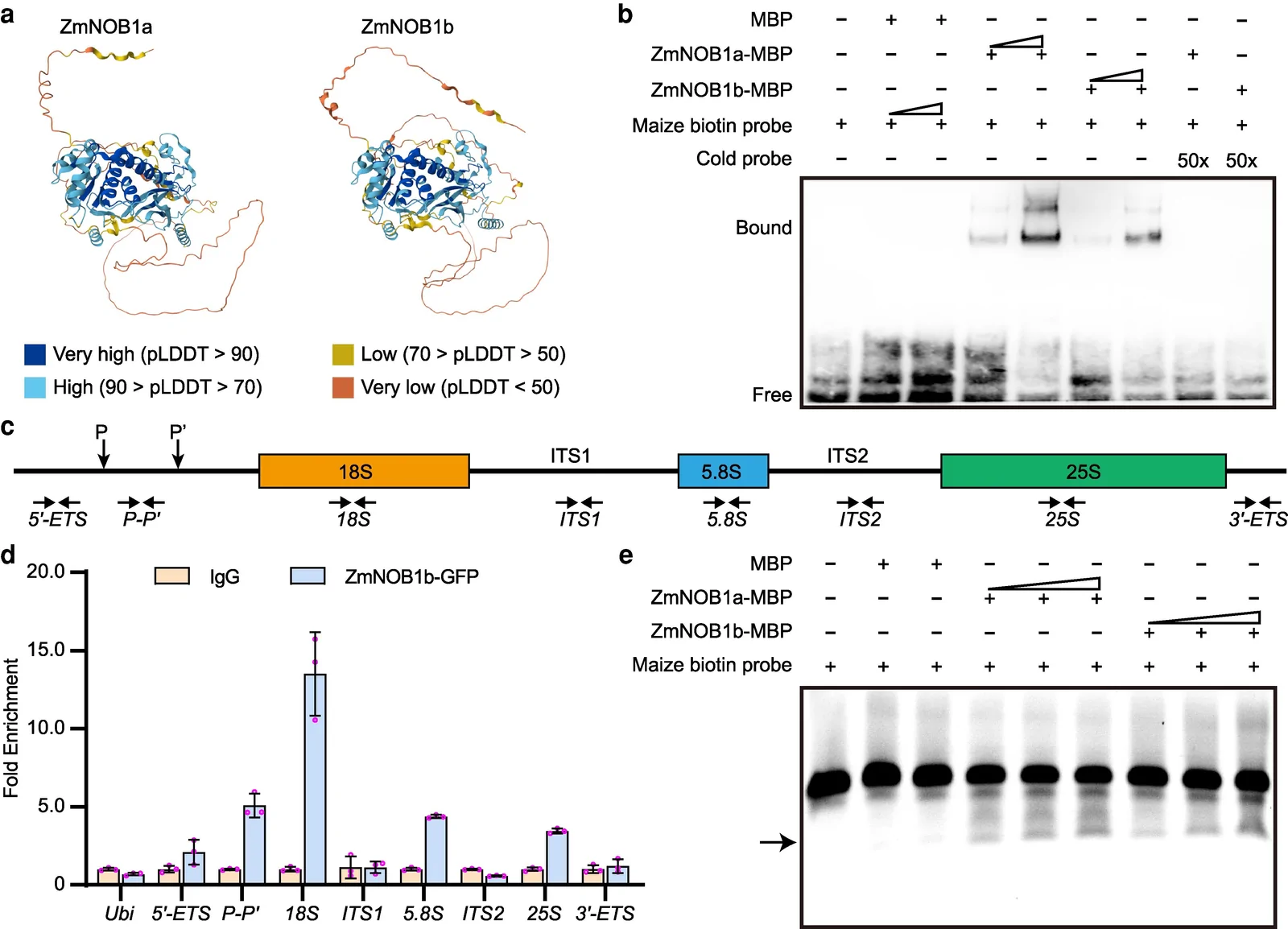
ZmNOB1a and ZmNOB1b specifically bind 18S pre-rRNA and possess endonuclease activity. a) Predicted structures of ZmNOB1a and ZmNOB1b generated by AlphaFold (https://www.alphafold.ebi.ac.uk/). Structural models are shaded according to prediction confidence, as indicated by the scale bar. b) EMSA showing binding of ZmNOB1a-MBP and ZmNOB1b-MBP fusion proteins to a biotin-labeled RNA probe corresponding to the 3′ region of maize 18S rRNA and the 5′ region of ITS1. Increasing amounts of recombinant proteins were incubated with the probe, followed by separation on a 6% native polyacrylamide gel. The concentration of the competitor is 50-fold that of the biotin-labeled probe. c) Schematic representation of maize 45S pre-rRNA. Arrows indicate the positions of primers used for RIP-qPCR analysis. d) RIP-qPCR analysis showing in vivo association of ZmNOB1b with pre-rRNA. RIP assays were performed using kernels expressing *proUbi::Flag-ZmNOB1b-GFP*. Relative enrichment of different pre-rRNA regions was quantified by qPCR. The maize *Ubi* locus was used as a negative control. Data represent means ± SD (*n* = 3 biological replicates). e) In vitro endonuclease assay showing cleavage of RNA substrates by ZmNOB1a-MBP and ZmNOB1b-MBP. Cleavage products were resolved by denaturing urea-PAGE and visualized by ethidium bromide staining.

To determine whether maize NOB1 proteins retain RNA-binding and catalytic activities, recombinant ZmNOB1a-MBP and ZmNOB1b-MBP proteins were purified from *Escherichia coli* (Figure S2a). Biotin-labeled RNA substrates encompassing the maize D-site region were synthesized for electrophoretic mobility shift assays (EMSAs) (Figure S2b). Both ZmNOB1a and ZmNOB1b bound the maize pre-rRNA substrate in a concentration-dependent manner, whereas the MBP control displayed no detectable interaction (Fig. 1b). Binding specificity was further supported by efficient competition with a 50-fold molar excess of unlabeled probe. In contrast, ZmNOB1a failed to recognize the corresponding yeast D-site substrate (Figure S2, c and d). Sequence comparison revealed pronounced divergence in cleavage-site composition and ITS1 length between maize and yeast pre-rRNAs (Figure S2e), potentially accounting for the observed substrate selectivity.

To assess in vivo association with pre-rRNA, RIP-qPCR assays were performed using developing kernels expressing *proUbi::3×Flag-ZmNOB1b-GFP*. Analysis of distinct regions within the 45S pre-rRNA revealed pronounced enrichment of 18S-containing fragments, with approximately 13-fold enrichment relative to the IgG control (Fig. 1, c and d), indicating preferential association of ZmNOB1 with 18S pre-rRNA intermediates in vivo. We next evaluated endonuclease activity using denaturing PAGE-based assays. Increasing amounts of recombinant ZmNOB1a-MBP or ZmNOB1b-MBP progressively enhanced cleavage-product accumulation from maize D-site RNA substrates, whereas MBP alone exhibited no detectable activity (Fig. 1e). Comparable results were obtained using extended substrates spanning the 5′ flanking region of site D (Figure S3, a and b). Notably, ZmNOB1a consistently displayed stronger cleavage activity than ZmNOB1b. Moreover, both proteins retained measurable catalytic activity in the absence of exogenous Mn^2+^, although cleavage efficiency was reduced (Figure S3b). Collectively, these results establish that maize NOB1 paralogs function as sequence-selective RNA-binding endonucleases capable of mediating D-site cleavage.

### ZmNOB1a and ZmNOB1b physically interact in the cytoplasm

RT-qPCR analyses revealed highly similar expression patterns for *ZmNOB1a* and *ZmNOB1b* across maize tissues, with strongest accumulation detected in metabolically active organs, including shoot, ear, embryo, and developing endosperm (Fig. 2a). This expression pattern is consistent with the elevated translational demand associated with rapid cellular proliferation and storage reserve synthesis.

**Figure 2:**
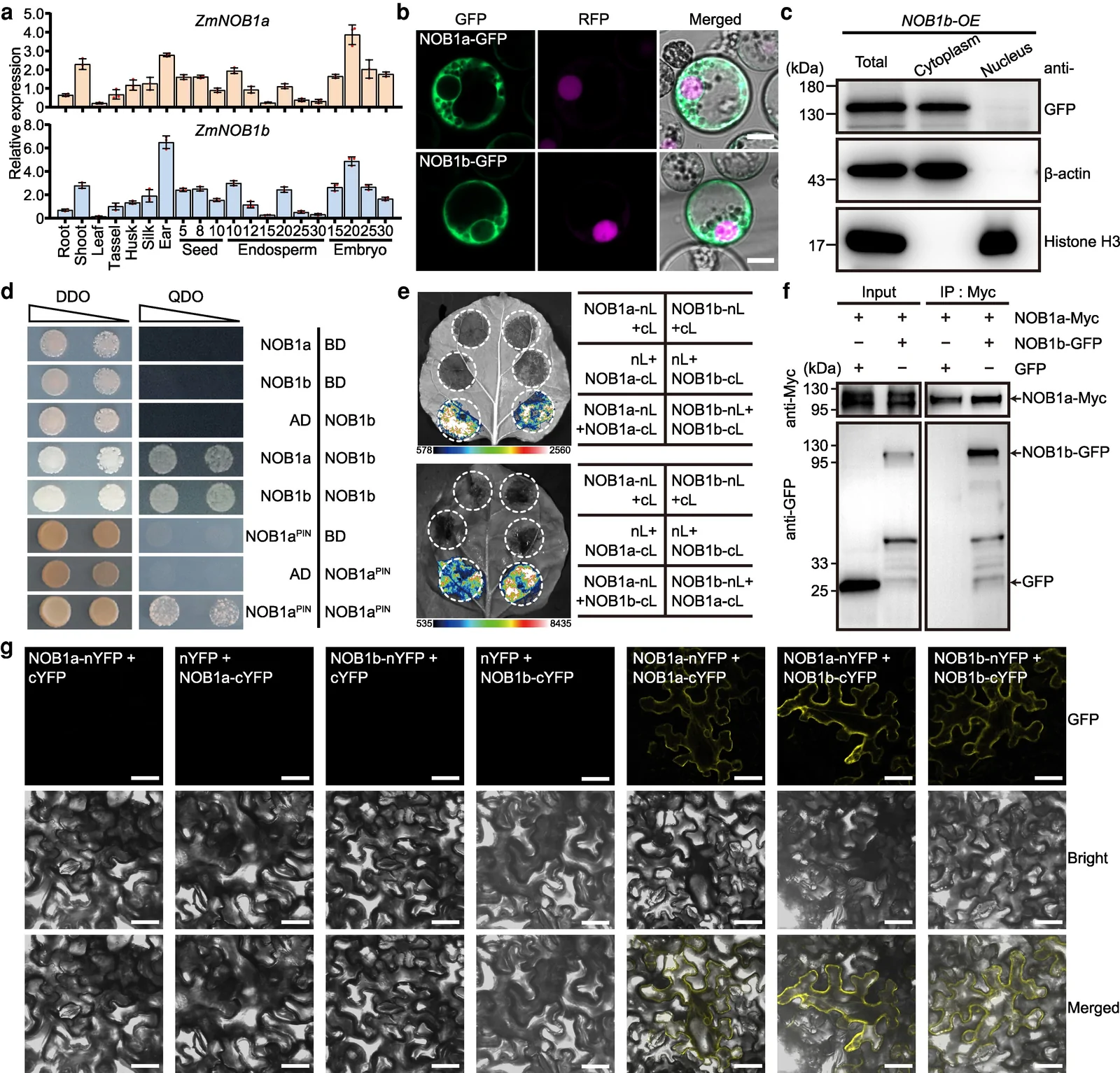
ZmNOB1a and ZmNOB1b interact with each other and localize predominantly to the cytoplasm. a) RT-qPCR analysis of *ZmNOB1a* and *ZmNOB1b* expression in different maize tissues. Transcript levels were normalized to *Ubi*. Numbers following tissue names indicate DAP. Data represent means ± SD (*n* = 3 biological replicates). Independent experiments using samples from different ears produced similar results. b) Subcellular localization of ZmNOB1 proteins in maize protoplasts. ZmNOB1-GFP fusion proteins were co-expressed with the nuclear marker RRP7-RFP. Comparable fluorescence patterns were observed in no fewer than 5 independent visual fields. Scale bar = 10 μm. c) Immunoblot analysis of ZmNOB1b in nuclear and cytoplasmic fractions isolated from 19-DAP kernels of *ZmNOB1b* overexpression plants. Anti-β-actin and anti-Histone H3 antibodies were used as cytoplasmic and nuclear markers, respectively. d to g) Interaction between ZmNOB1a and ZmNOB1b revealed by Y2H (d), LCI (e), Co-IP (f), and BiFC (g) assays. Scale bar = 40 μm.

In yeast and animals, NOB1-mediated cleavage of 20S pre-rRNA occurs following cytoplasmic export of pre-40S particles and serves as a critical checkpoint during ribosome maturation (Cerezo et al. 2019). To determine the localization of maize NOB1 proteins, GFP-tagged constructs were transiently expressed in *Nicotiana benthamiana* epidermal cells and maize protoplasts. Both ZmNOB1a-GFP and ZmNOB1b-GFP accumulated predominantly in the cytoplasm (Fig. 2b; Figure S4). Consistently, immunoblot analysis of fractionated extracts from 19 days after pollination (DAP) kernels expressing *proUbi::3×Flag-ZmNOB1b-GFP* demonstrated preferential enrichment of ZmNOB1 in the cytoplasmic fraction (Fig. 2c).

Structural analyses in yeast previously showed that NOB1 assembles into oligomeric complexes through its PIN domain (Lamanna and Karbstein 2009). To determine whether maize NOB1 paralogs similarly associate with one another, we performed multiple protein–protein interaction assays. Yeast two-hybrid (Y2H) analysis showed that ZmNOB1a and ZmNOB1b engage in both homotypic and heterotypic interactions, primarily mediated by the PIN domain (Fig. 2d). These interactions were independently validated in planta using luciferase complementation imaging (LCI), co-immunoprecipitation (Co-IP), and bimolecular fluorescence complementation (BiFC) assays (Fig. 2, e to g). Together, these results indicate that ZmNOB1a and ZmNOB1b assemble into homo- and heteromeric complexes within the cytoplasm.

### Single mutations in *ZmNOB1* genes exert minimal effects on pre-rRNA maturation

Two EMS-induced nonsense alleles were obtained for each of *ZmNOB1a* and *ZmNOB1b* in the B73 genetic background. Following repeated backcrossing, 4 homozygous mutant lines (*aa-1*, *aa-2*, *bb-1*, and *bb-2*) were obtained. All alleles introduced premature stop codons near the N-terminus; notably, the *bb-2* allele lacks the entire PIN domain, whereas the remaining alleles retain the PIN domain but lack the Zn-ribbon domain (Fig. 3a). All 4 mutants were viable and exhibited 100-kernel weights comparable to B73 (Fig. 3, b and c). However, seed set was significantly reduced in the *bb-1* and *bb-2* mutants (Fig. 3b). Quantitative morphometric analysis revealed that while *ZmNOB1a* mutations did not alter mature kernel length or width, *ZmNOB1b* mutations significantly modified kernel aspect ratio by decreasing length while increasing width and thickness (Figure S5). Histological analysis further showed normal embryo and endosperm organization, including proper differentiation of the scutellum (SL), coleoptile (Co), shoot apical meristem (SAM), and root primordium (Rp) (Figure S6). These observations indicate substantial functional redundancy between *ZmNOB1a* and *ZmNOB1b*, whereas the reproductive defects observed in *zmnob1b* mutants suggest partial subfunctionalization following gene duplication.

**Figure 3:**
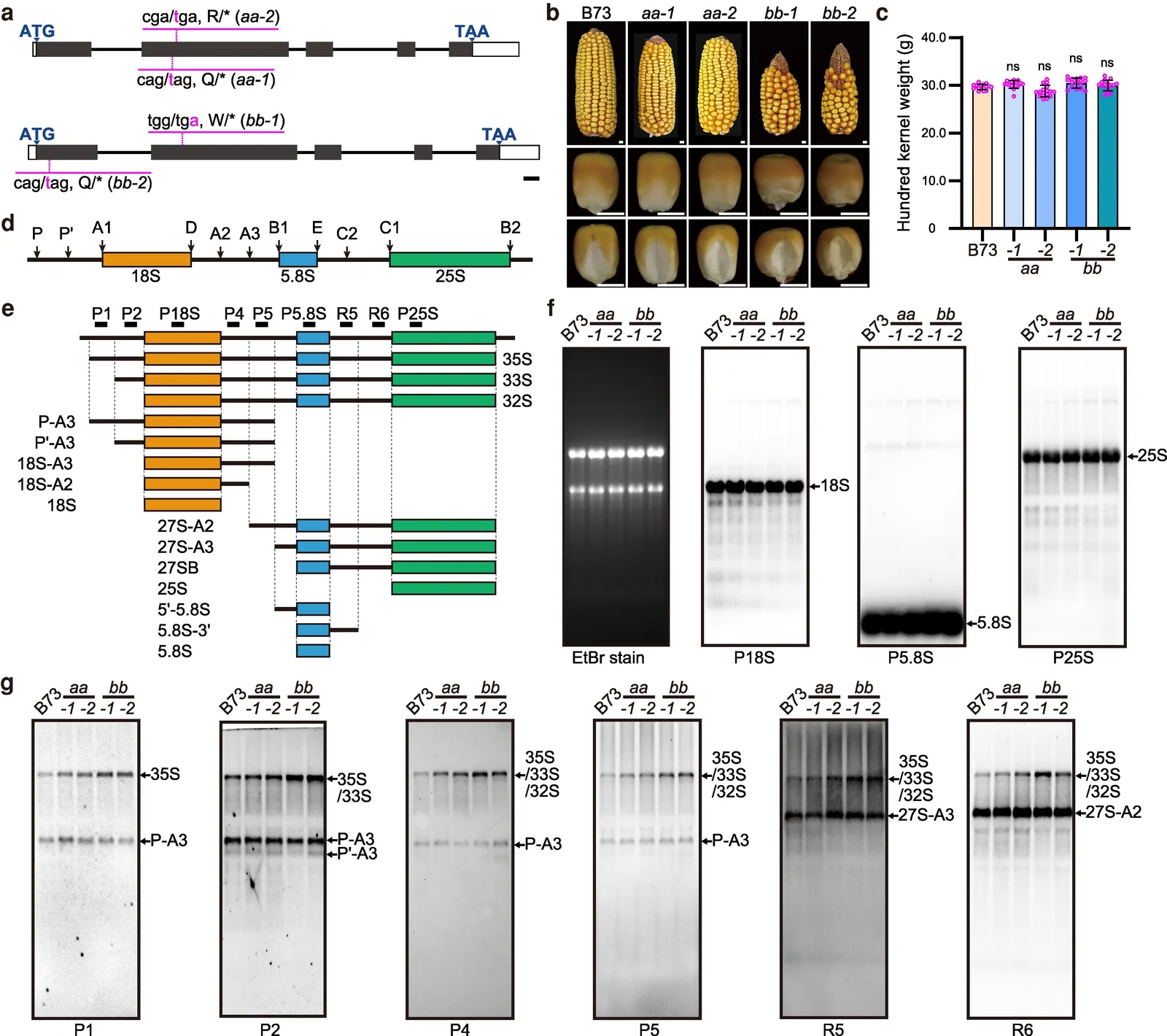
Single mutants of *zmnob1a* or *zmnob1b* do not exhibit detectable defects in pre-rRNA processing. a) Gene structures of *ZmNOB1a* and *ZmNOB1b*. Boxes and connecting lines indicate exons and introns, respectively. The lateral lines mark EMS-induced premature stop codons. Scale bar = 100 bp. b) Mature ears and kernels harvested from self-pollinated B73 and homozygous *zmnob1* mutant plants. Scale bar = 50 mm. c) Hundred-kernel weight of B73 and *zmnob1* mutants. Data were obtained from 3 independently self-pollinated homozygous ears and are shown as means ± SD (*n* = 16). Statistical significance was determined by one-way ANOVA relative to B73 (ns, not significant). d) Schematic representation of the maize 45S pre-rRNA processing pathway. Arrows indicate major cleavage sites. e) Schematic diagram of pre-rRNA processing intermediates and positions of probes used for RNA gel-blot analysis. Probe positions are indicated by short lines. f) RNA gel-blot analysis of mature rRNAs using probes specific for P18S, P5.8S, and P25S. Total RNA was isolated from 19-DAP kernels. Equal amounts of RNA were separated on denaturing formaldehyde-agarose gels. Ethidium bromide staining served as a loading control. Mature rRNA species are indicated on the right. g) RNA gel-blot analysis of pre-rRNA intermediates using probes P1, P2, P4, R5, and R6. Pre-rRNA intermediates are indicated on the right.

To determine whether individual mutations affect pre-rRNA processing, we analyzed rRNA intermediates from 19-DAP kernels by northern blotting using probes spanning the maize 45S pre-rRNA transcript (Fig. 3, d and e). Accumulation of mature 18S, 5.8S, and 25S rRNAs remained comparable between B73 and mutant kernels (Fig. 3f), indicating that disruption of either paralog alone is insufficient to compromise global rRNA maturation. Nevertheless, probes P1, P2, P4, P5, R5, and R6 detected moderate accumulation of the 35S precursor in mutant kernels, whereas downstream intermediates, including P′-A3, 27S-A2, and 27S-A3 remained largely unaffected (Fig. 3g). Thus, single *ZmNOB1* mutations exert only limited effects on pre-rRNA processing.

To further evaluate rRNA maturation, circular RT-PCR analyses were performed. The levels of 35S and 32S precursors remained essentially unchanged in the mutant kernels (Figure S7, a and b). Amplification of multiple 18S precursor species revealed no evident alterations in the accumulation of P-A3, 18S-A3, or 18S-A2 intermediates relative to B73 (Figure S7, c and d). Because the CR2/R8 primer pair amplifies both mature 18S rRNA and 20S precursors, PCR products were cloned and sequenced to evaluate D-site processing fidelity. Among 19, 15, and 17 clones analyzed from B73, *aa-1*, and *bb-1*, respectively, only a single aberrant cleavage event was detected in each genotype (Figure S8). In addition, no apparent defects were detected in 5.8S or 25S precursor processing (Figure S7, e to h). Collectively, these findings demonstrate that *ZmNOB1a* and *ZmNOB1b* function redundantly during maize pre-rRNA maturation.

### Functional redundancy between *ZmNOB1a* and *ZmNOB1b* is indispensable for maize development

To assess the extent of functional redundancy between the 2 maize NOB1 paralogs, we crossed homozygous *zmnob1a* and *zmnob1b* mutants to generate 4 double-heterozygous combinations. Self-pollinated ears from these plants segregated kernels displaying severe developmental abnormalities (Fig. 4a). Genotyping demonstrated that the defective kernels recovered from *Aa1Bb1* ears corresponded to the *Aa1b1b1* genotype (Figure S9, a to c). Although Mendelian inheritance predicts the occurrence of double-homozygous *aabb* progeny, no such kernels were identified among the offspring, suggesting that transmission of the *ab-*carrying male gametophyte is severely compromised. Consistent with this interpretation, I_2_-KI staining revealed that approximately one-quarter of pollen grains from *AaBb* plants failed to stain, whereas pollen from B73 exhibited uniform staining and normal morphology (Fig. 4, b and c). To further examine gamete transmission efficiency, we performed reciprocal crosses. When *AaBb* plants served as the male parent in crosses with B73, no *AaBb* progeny were detected by genotyping, confirming that the *ab* haplotype is not transmitted through the male gametophyte and that male gametophytes carrying *ab* completely lose their transmission capacity. When *AaBb* plants served as the female parent crossed with B73, a total of 1,152 kernels were genotyped, among which only 126 were identified as *AaBb*, representing a frequency significantly lower than the expected Mendelian ratio. This indicates that transmission through the female gametophyte is also severely impaired (Fig. 4d; Table S1). Collectively, these results demonstrate that *ZmNOB1a* and *ZmNOB1b* function redundantly to sustain gametophyte viability.

**Figure 4:**
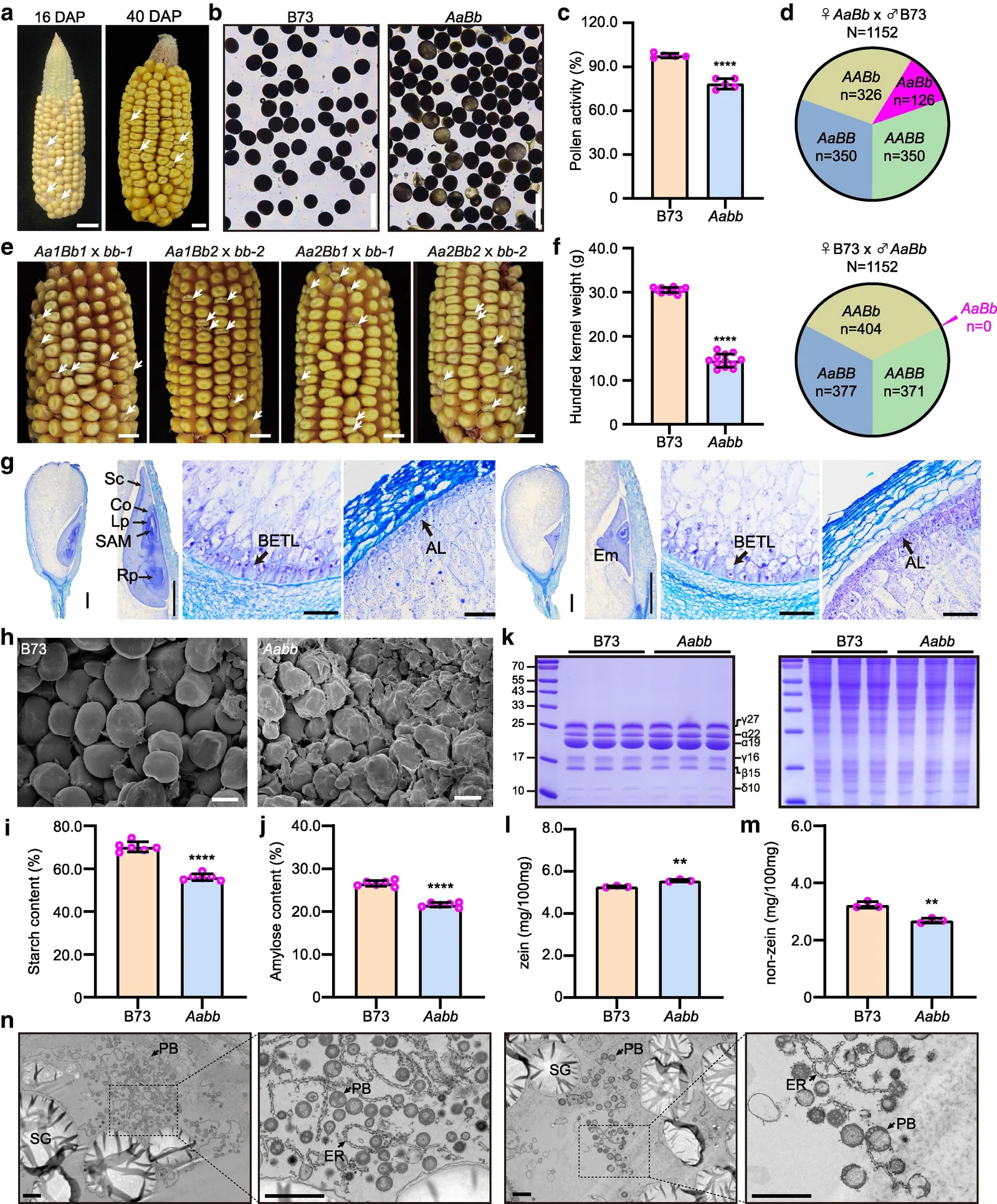
The *Aabb* mutant exhibits impaired pollen viability and defective kernel development. a) Ear phenotypes of self-pollinated *AaBb* plants. Arrows mark defective kernels with the *Aabb* genotype. Scale bar = 1 cm. b and c) Pollen viability of B73 and *AaBb* plants assessed by I_2_-KI staining. Representative micrographs are presented in (b), and quantification data are shown in (c). Data represent means ± SD (*n* = 5 biological replicates). Statistical significance was determined by two-tailed Student's *t*-test (****P < 0.0001). Scale bar = 200 μm. d) Genotyping of progeny kernels from reciprocal crosses between B73 and *AaBb* plants using dCAPS markers. e) Mature ears obtained from crosses where *AaBb* served as the female parent and homozygous *zmnob1b* mutants as the pollen donor. Arrows indicate defective kernels. Scale bar = 1 cm. f) Hundred-kernel weight of mature B73 and *Aabb* kernels. Data represent means ± SD (*n* = 12). Statistical significance was determined by a two-tailed Student's *t*-test (****P < 0.0001). g) Longitudinal paraffin sections of B73 and *Aabb* kernels at 17 DAP. Enlarged views of the embryo, BETL, and aleurone layer (AL) are shown. Scale bars = 1 mm (embryo) and 100 μm (BETL and AL). Lp, leaf primordia. h) Scanning electron micrographs of starch granules in the central endosperm of mature B73 and *Aabb* kernels. Scale bar = 10 μm. i and j) Total starch (i) and amylose (j) contents in mature kernels of B73 and *Aabb*. Data represent means ± SD (*n* = 6). Statistical significance was determined by two-tailed Student's *t*-test (****P < 0.0001). k) SDS-PAGE analysis of zein and non-zein proteins extracted from mature B73 and *Aabb* kernels. Molecular weight markers are indicated on the left, and zein subfamilies are labeled on the right. Three biological replicates are shown. l and m) Quantification of zein (l) and non-zein (m) protein contents in mature kernels. Data represent means ± SD (*n* = 3 biological replicates). Statistical significance was determined by a two-tailed Student's *t*-test (**P < 0.01). n) Transmission electron micrographs of endosperm cells isolated from B73 and *Aabb* kernels at 19-DAP. Insets show enlarged views of boxed regions. Scale bar = 5 μm. ER, endoplasmic reticulum.

Crosses between *AaBb* and *AAbb* plants yielded ears segregating visibly shrunken kernels corresponding to the *Aabb* genotype (Fig. 4e). Mature *Aabb* kernels exhibited dramatic reductions in kernel size and 100-kernel weight relative to B73 (Fig. 4f; Figure S9, d to i). In addition, *Aabb* plants displayed dwarfism and aberrant tassel architecture (Figure S9, j and k), indicating that partial loss of ZmNOB1 activity broadly compromises vegetative and reproductive development.

Histological examination of 17-DAP kernels revealed a profound delay in embryo development in *Aabb* mutants (Fig. 4g). The basal endosperm transfer layer (BETL) failed to differentiate properly and displayed severe cellular abnormalities, whereas aleurone cells appeared densely packed and disorganized (Fig. 4g). Scanning electron microscopy further showed that starch granules (SG) in *Aabb* endosperm were markedly smaller and morphologically irregular compared with the smooth and spherical granules observed in B73 (Fig. 4h). Consistent with these structural defects, both total starch and amylose contents were significantly reduced in *Aabb* kernels (Fig. 4, i and j). SDS-PAGE assays revealed increased zein accumulation accompanied by a substantial reduction in non-zein proteins in *Aabb* mature kernels (Fig. 4k), which was corroborated by quantitative protein measurements (Fig. 4, l and m). Transmission electron microscopy observation additionally uncovered a striking decrease in protein body (PB) abundance together with severe abnormalities in protein body morphology within *Aabb* endosperm cells (Fig. 4n). These findings indicate that ZmNOB1 activity is critical for proper endosperm differentiation and coordinated accumulation of storage reserves during kernel development.

Interestingly, crosses between *AaBb* and *aaBB* plants recovered viable small-kernel progeny carrying the *aaBb* genotype (Figure S10, a and b). Although *aaBb* kernels displayed significant reductions in kernel size and weight relative to B73 (Figure S10, c to i), their developmental defects were consistently less severe than those observed in *Aabb.* These observations suggest that ZmNOB1b contributes more prominently than ZmNOB1a to kernel development. Moreover, no defective kernels were recovered when *AaBb* plants were used as the pollen donor in crosses with either *aaBB* or *AAbb* plants (Figure S10j), further supporting defective transmission of the male gametophyte carrying the *ab* haplotype.

### Simultaneous disruption of ZmNOB1a and ZmNOB1b perturbs multi-step pre-rRNA processing

To investigate how combined loss of ZmNOB1 activity influences ribosome biogenesis, we quantified pre-rRNA intermediates in 19-DAP *Aabb* and *aaBb* kernels by RT-qPCR. In *Aabb*, transcripts corresponding to intermediates containing the 5′-ETS, 18S, ITS1, 5.8S, ITS2, and 25S sequences accumulated to substantially higher levels than in B73, whereas the P–P′ fragment remained largely unchanged and 3′-ETS decreased (Fig. 5a). By contrast, *aaBb* kernels displayed comparatively modest increases in 18S-containing intermediates, whereas ITS-containing fragments showed an overall declining trend (Fig. 5a). These results indicate that ribosome biogenesis is disrupted in both mutant backgrounds, with more severe defects in *Aabb*.

**Figure 5:**
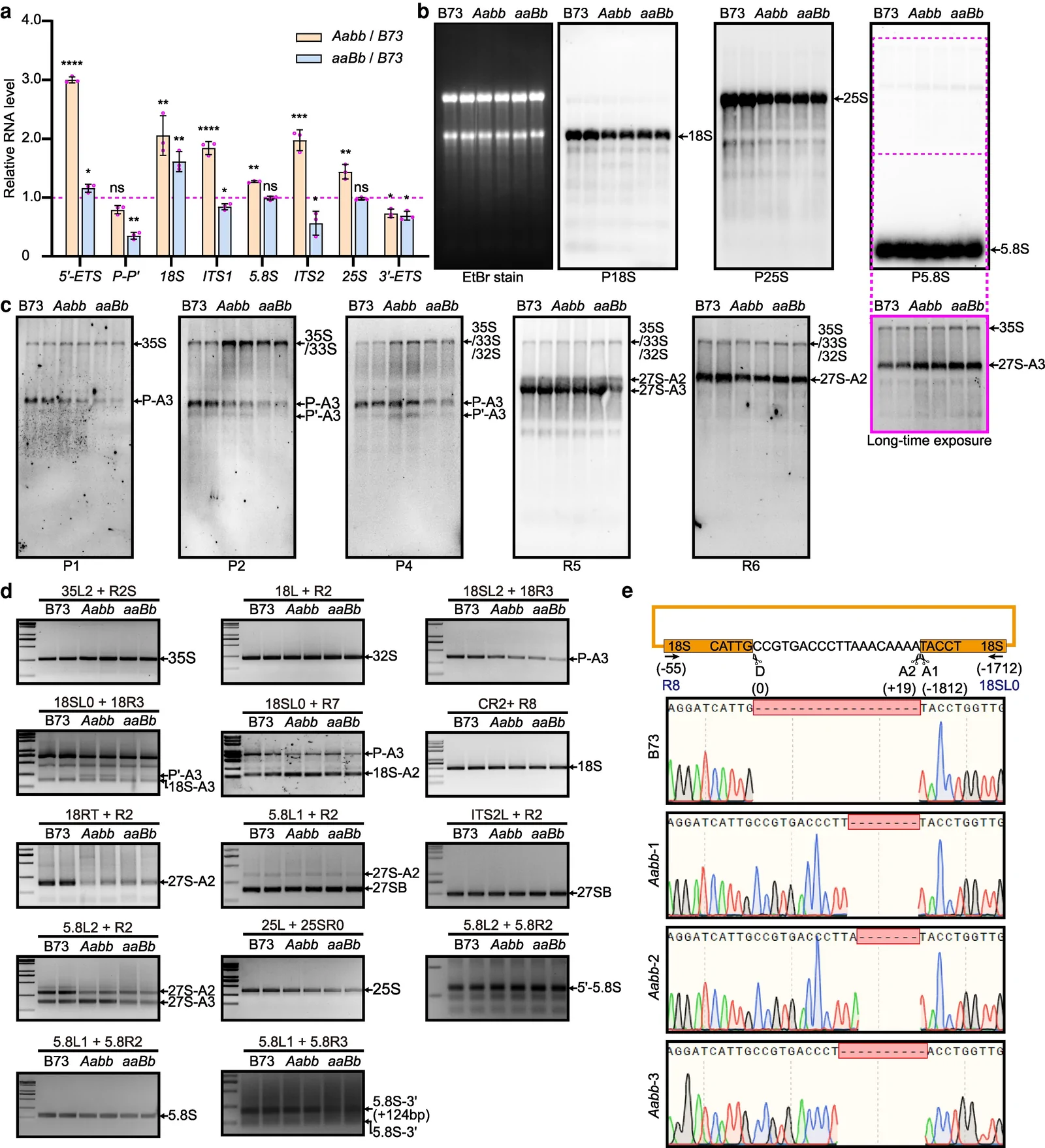
Pre-rRNA processing is disrupted in *Aabb* and *aaBb* mutant kernels. a) RT-qPCR analysis of pre-rRNA accumulation in *Aabb* and *aaBb* kernels relative to B73. Total RNA was isolated from 19-DAP kernels. Expression levels were normalized to *Ubi*. Data represent means ± SD (*n* = 3 biological replicates). Statistical significance was determined by a two-tailed Student's *t*-test (ns, not significant; *P < 0.05; **P < 0.01; ***P < 0.001; ****P < 0.0001). b) RNA gel-blot analysis of mature rRNAs using probes specific for P18S, P5.8S, and P25S in B73, *Aabb*, and *aaBb* kernels at 19 DAP. Equal amounts of RNA were separated on denaturing formaldehyde-agarose gels. Ethidium bromide staining served as a loading control. Positions of rRNA species are indicated on the right. Inset boxes indicate longer exposure images showing 35S and 27S-A3 intermediates. c) RNA gel-blot analysis of pre-rRNA intermediates in B73, *Aabb*, and *aaBb* using probes P1, P2, P4, R5, and R6. d) Mapping of 5′ and 3′ termini of pre-rRNA intermediates in B73, *Aabb*, and *aaBb* by circular RT-PCR. e) Defective D-site cleavage in *Aabb* mutant kernels. Sequencing analysis of the shifted PCR products shown in Figure S11.

Northern blot analyses further revealed pronounced reductions in mature 18S and 25S rRNA accumulation in both *Aabb* and *aaBb* kernels, whereas 5.8S rRNA levels were largely unchanged (Fig. 5b). These findings suggest that ZmNOB1 function is not restricted to D-site cleavage during 18S rRNA maturation, but additionally indirectly contributes to processing events associated with large-subunit biogenesis. Consistent with this notion, multiple pre-rRNA intermediates including 35S, P′-A3, and 27S-A3 accumulated strongly in both mutants, particularly in *Aabb*, whereas P-A3 and 27S-A2 levels were markedly reduced (Fig. 5c). Because probe P2 recognizes both 35S and 33S precursors, the stronger P2 signal relative to P1 likely reflects enhanced accumulation of the 33S intermediate. Circular RT-PCR analyses further confirmed elevated 35S precursor levels in the mutants (Fig. 5d).

To dissect defects associated with 18S maturation, we amplified distinct processing intermediates using multiple primer combinations (Figure S7c). Amplification with 18SL0/18R3 revealed strong accumulation of the P′-A3 precursor accompanied by reduced 18S-A3 abundance, indicative of impaired cleavage at site A1 (Fig. 5d). In parallel, amplification using 18SL0/R7 showed pronounced accumulation of the 18S-A2 precursor, suggesting a defect in D-site cleavage. Because the CR2/R8 primer pair amplifies both mature 18S rRNA and 18S-A2 fragments of similar size, PCR products were cloned and sequenced to discriminate between the 2 species. However, only correctly processed mature 18S rRNA was detected in B73; aberrantly processed products accumulated in *Aabb* mutant kernels (Fig. 5e; Figure S11), confirming severe disruption of D-site processing.

We next examined processing events associated with 25S maturation. Quantitative analysis of 27S precursor intermediates revealed a slight accumulation of 27S-A3 alongside a marked reduction in 27S-A2 levels in *Aabb* mutants, whereas 27SB levels remained unchanged (Fig. 5d; Figure S7e). Consistent with the reduced accumulation of mature 25S rRNA, these data suggest that processing at sites B1 and C1 is affected in the mutants. Moreover, increased accumulation of the 5′-5.8S precursor, as detected by the 5.8L2/5.8R2 primer pair, further supports impaired processing at the B1 site (Fig. 5d). Collectively, these results demonstrate that simultaneous disruption of ZmNOB1a and ZmNOB1b impairs cytoplasmic D-site cleavage of 18S pre-rRNA and indirectly alters processing efficiency at sites A1, B1, and C1.

### ZmNOB1 associates with pre-40S ribosomal maturation complexes

In yeast and animals, NOB1 functions as a component of late cytoplasmic pre-40S ribosomal particles together with multiple ribosome assembly factors (Fatica et al. 2003; Ameismeier et al. 2020). To determine whether maize NOB1 proteins similarly associate with conserved pre-40S maturation components, we examined interactions between ZmNOB1 proteins and several candidate assembly factors, including Pno1, Enp1, Rps5, and Rio2 (Data Set S3). Among these, *Pno1* was found to have 2 paralogous genes (*Pno1-1* and *Pno1-2*). Protein interaction assays revealed a strong physical interaction between ZmNOB1 and Pno1, whereas no detectable interaction was observed with Enp1, Rps5, or Rio2 in Y2H assays (Fig. 6a). We further confirmed these physical interactions in *N. benthamiana* using Co-IP and LCI assays. Co-IP assays demonstrated that both ZmNOB1a and ZmNOB1b interact with Pno1, Enp1, Rps5, and Rio2 in vivo (Fig. 6b). Consistent with these results, strong luminescence was observed in LCI assays upon co-expressed ZmNOB1-nLUC with Pno1-, Enp1-, Rps5-, and Rio2-cLUC constructs, confirming that ZmNOB1 stably associates with these 40S subunit assembly factors (Fig. 6c). Collectively, these results demonstrate that maize NOB1 proteins associate with evolutionarily conserved pre-40S ribosomal maturation complexes.

**Figure 6:**
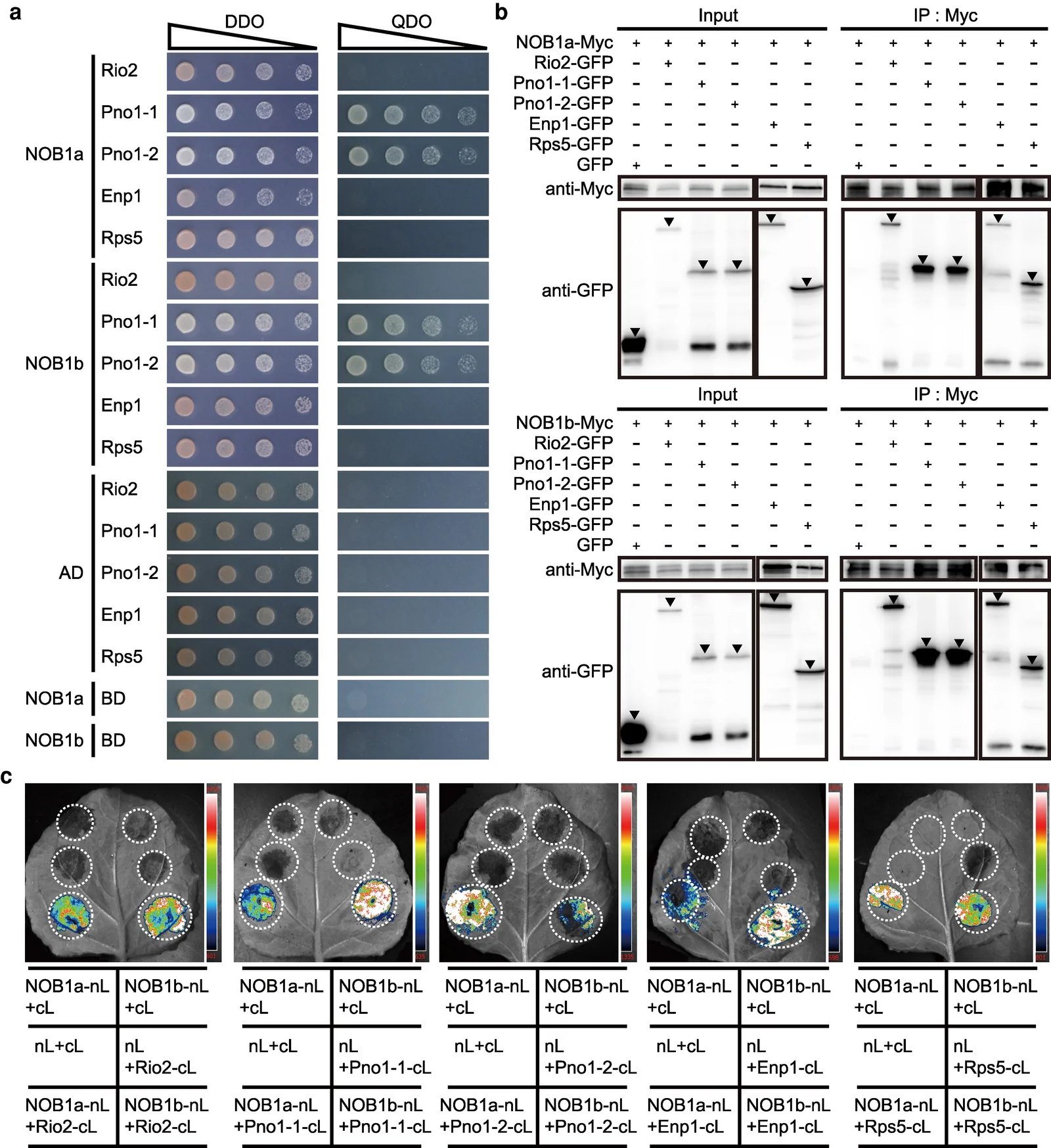
ZmNOB1 proteins associate with multiple pre-40S ribosome assembly factors. a) Y2H assays showing interactions between ZmNOB1 proteins and putative pre-40S assembly factors, including ZmRio2, ZmPno1-1, ZmPno1-2, ZmEnp1, and ZmRps5. 5-fold serial dilutions of yeast cultures are shown. b) Co-IP assays confirming interactions between ZmNOB1 proteins and pre-40S assembly factors. Immunoblots of input and Myc-immunoprecipitated samples were detected using anti-GFP and anti-Myc antibodies, respectively. Triangles indicate target proteins. c) LCI assays showing interactions between ZmNOB1 proteins and pre-40S assembly factors in *N. benthamiana* leaves.

### Mutant kernels exhibit impaired ribosome assembly and translational competence

During translation initiation, the 40S ribosomal subunit is recruited to the 5′ terminus of mRNA, scans toward the initiation codon, and subsequently joins with the 60S subunit to assemble a translationally competent 80S ribosome. To determine whether impaired pre-rRNA processing compromises translation in *zmnob1* mutants, we analyzed ribosome profiles from 19-DAP kernels by sucrose density-gradient centrifugation. Both *Aabb* and *aaBb* mutants displayed marked accumulation of free 40S and 60S subunits, accompanied by substantial reductions in 80S monosomes and polysomes (Fig. 7a). Immunoblotting demonstrated that NOB1 was predominantly enriched in the initiation fractions and strongly co-fractionated with 40S subunits in the mutants. Meanwhile, RPS6 and RPL13 accumulated more in the subunit fractions but decreased in the monosome fractions (Fig. 7a). Quantification of the polysome-to-non-polysome ratio further revealed a dramatic decline in translationally active ribosomes in both mutant backgrounds (Fig. 7b). We next examined the accumulation of additional ribosome biogenesis factors (RCL1 and PUM24) and ribosomal proteins (RPS6 and RPL13). Protein levels of all 4 candidates were higher in mutants than in B73 (Fig. 7c), aligning with the elevated free subunit pool observed in the polysome profiles. At the transcriptional level, RT-qPCR analysis showed significant upregulation of multiple ribosomal protein genes in *Aabb*, whereas most remained unchanged in *aaBb* (Figure S12). Together, these results indicate that loss of ZmNOB1 function impairs ribosome assembly and broadly attenuates translational capacity.

**Figure 7:**
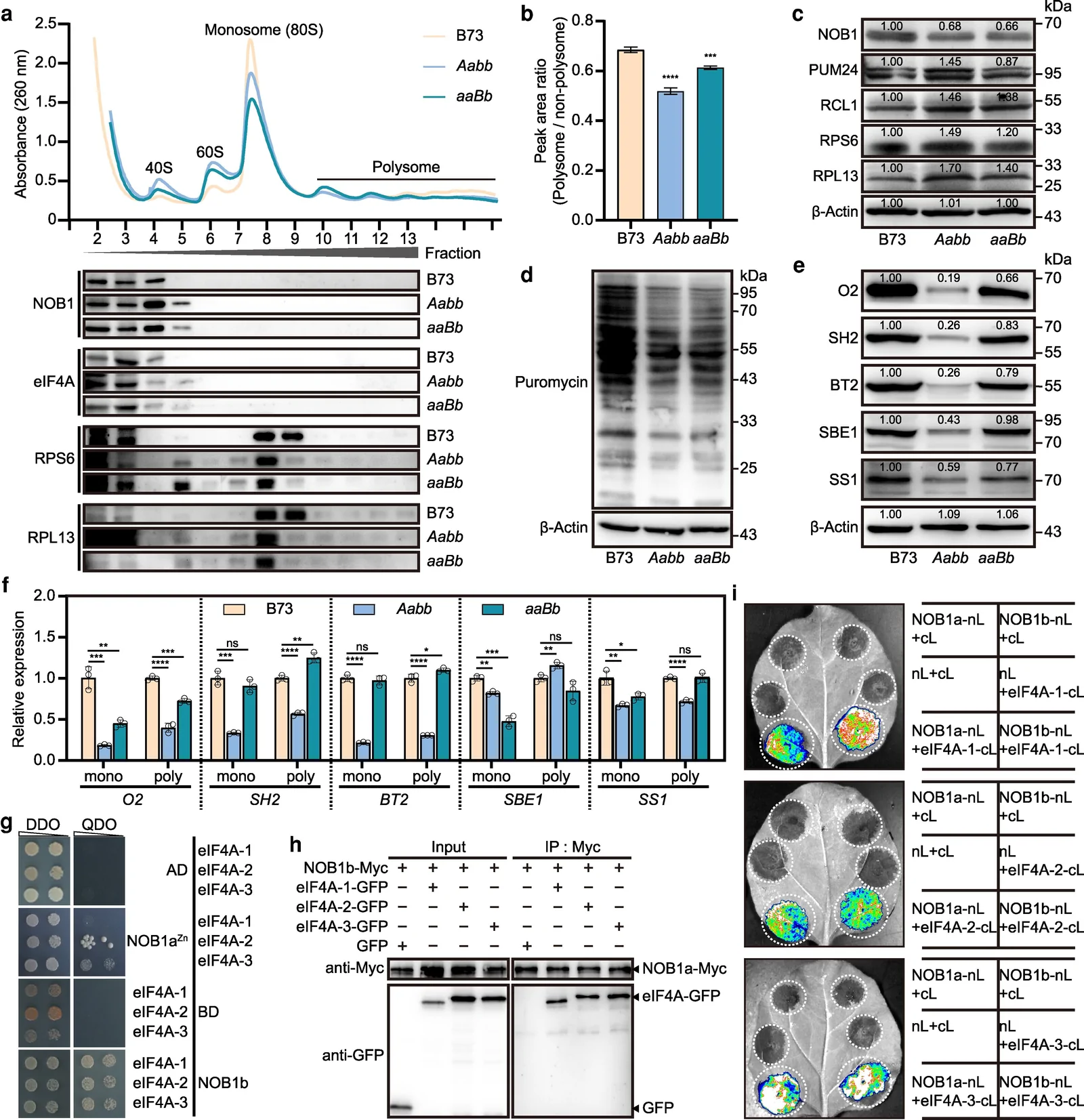
Ribosome assembly and translational activity are compromised in *Aabb* and *aaBb* mutants. a) Polysome profiles of B73, *Aabb*, and *aaBb* kernels at 19 DAP. Ribosome complexes were separated through 10% to 45% sucrose gradients and monitored by absorbance at 260 nm. Peaks corresponding to 40S, 60S, monosome (80S), and polysomes are indicated. Fractions from sucrose gradients were collected and subjected to western blot analysis. b) Quantification of the polysome-to-non-polysome ratio from ribosome profiles shown in (a). Data represent means ± SD. Statistical significance was determined by two-tailed Student's *t*-test (***P < 0.001; ****P < 0.0001). c) Immunoblot analysis of the RBFs and RPs in B73, *Aabb*, and *aaBb*. Total proteins were extracted from 19-DAP kernels. β-Actin served as a loading control. d) Immunoblot detection of puromycin incorporation into nascent polypeptides in seedlings of B73, *Aabb*, and *aaBb*, indicating global translational activity. β-Actin served as a loading control. e) Immunoblot analysis of O2, SH2, BT2, SBE1, and SS1 protein accumulation in 19-DAP kernels of B73, *Aabb*, and *aaBb*. β-Actin served as a loading control. f) The abundance of *O2*, *SH2*, *BT2*, *SBE1*, and *SS1* mRNAs associated with monosomes and polysomes was examined using RT-qPCR. RNA was extracted from monosome and polysome fractions collected by sucrose density-gradient centrifugation. Expression levels were normalized to *Ubi*. Data represent means ± SD (*n* = 3 biological replicates). Statistical significance was determined by two-tailed Student's *t*-test (ns, not significant; *P < 0.05; v**P < 0.01; ***P < 0.001; ****P < 0.0001). g to i) Interactions between ZmNOB1 and eIF4A were examined by Y2H (g), Co-IP (h), and LCI (i) assays.

To directly evaluate protein synthesis, we performed puromycin incorporation assays in developing seedlings. Consistent with the polysome profiling results, puromycin labeling was markedly reduced in both *Aabb* and *aaBb* relative to B73, indicative of severely compromised global translational activity (Fig. 7d). Given the severe reduction in starch and non-zein protein accumulation observed in *Aabb* kernels (Fig. 4), we next examined the abundance of key regulators and enzymes involved in storage reserve biosynthesis. Immunoblot analyses revealed strongly reduced accumulation of Opaque2 (O2), SH2, BT2, SBE1, and SS1 proteins in *Aabb* kernels (Fig. 7e). Transcriptomic profiling further revealed widespread downregulation of genes encoding zein storage proteins and starch biosynthetic enzymes (Figure S13). Consistently, polysome profiling demonstrated that transcripts corresponding to these pathways exhibited substantially reduced association with both monosomes and polysomes (Fig. 7f). These observations suggest that defective storage reserve accumulation in *Aabb* primarily results from a profound reduction in translational output.

In eukaryotes, efficient recruitment of the 43S pre-initiation complex to capped mRNAs depends on the eIF4F complex. Intriguingly, both ZmNOB1 and eIF4A co-fractionated predominantly with the 40S subunit, with minimal signal in 60S, monosome, or polysome fractions (Fig. 7a). Subcellular localization confirmed that ZmNOB1 and eIF4A co-localize in the cytoplasm (Figure S14). To investigate whether ZmNOB1 additionally contributes to translation initiation, we tested its interaction with several eIF4F components. Y2H, Co-IP, and LCI assays demonstrated that ZmNOB1 directly interacts with the RNA helicase eIF4A (Fig. 7, g to i) and associates with the eIF4F complex in vivo (Figure S15). These findings raise the intriguing possibility that translational defects in *zmnob1* mutants arise from combined disruption of late-stage ribosome maturation and impaired recruitment of the translation pre-initiation machinery.

## Discussion

### Conserved but functionally diversified roles of duplicated maize NOB1 paralogs

NOB1 is an evolutionarily conserved endonuclease that catalyzes the terminal D-site cleavage required for cytoplasmic maturation of 18S rRNA in yeast, animals, and plants (Fatica et al. 2003; Ameismeier et al. 2018). Our biochemical analyses demonstrate that both ZmNOB1a and ZmNOB1b specifically recognize maize pre-rRNA substrates harboring the D-site and retain robust endonucleolytic activity in vitro (Figs. 1 and 5), indicating that the core catalytic function of NOB1 is maintained in maize. Nevertheless, ZmNOB1 proteins failed to recognize the corresponding yeast pre-rRNA substrate (Figure S2), suggesting that although the catalytic mechanism of NOB1 is evolutionarily conserved, and substrate recognition has undergone substantial species-specific divergence. Similar incompatibility has been reported for other pre-rRNA processing factors, including ZmRCL1, which is unable to complement the corresponding yeast mutant (Wang et al. 2022a). These observations suggest that conserved ribosome biogenesis factors have co-evolved with species-specific pre-rRNA architectures to ensure processing fidelity and structural compatibility during ribosome biogenesis.

Unlike yeast and Arabidopsis, which contain a single essential *NOB1* gene, maize contains 2 highly conserved paralogs. Our genetic analyses demonstrate that *ZmNOB1a* and *ZmNOB1b* function redundantly to sustain gametophyte viability, embryogenesis, and kernel development. However, single mutants remained viable and exhibited only modest developmental abnormalities; simultaneous reduction of both paralogs resulted in severe reproductive and profound developmental impairment (Figs. 3 and 4). Such redundancy likely provides developmental robustness during maize kernel development, a process characterized by exceptionally rapid growth and intense metabolic activity (Dai et al. 2021).

Despite their extensive functional overlap, the 2 paralogs are not fully equivalent. Mutations in *ZmNOB1b* produced stronger defects in seed set and ear development than mutations in *ZmNOB1a*, and the *Aabb* genotype consistently exhibited more severe developmental and molecular phenotypes than *aaBb* (Fig. 4; Figure S9). These findings suggest that the duplicated paralogs have undergone partial functional diversification following gene duplication. Such unequal redundancy likely allows maize to preserve the essential function of late ribosome maturation while simultaneously enabling developmental fine-tuning under distinct physiological contexts.

### ZmNOB1 defines a late-stage quality-control checkpoint during pre-40S maturation

Late maturation of the 40S ribosomal subunit constitutes a critical quality-control checkpoint during eukaryotic ribosome biogenesis. In yeast and human cells, NOB1 associates with pre-40S particles during nuclear maturation but remains catalytically inactive until export to the cytoplasm, where cleavage at the D-site ultimately generates mature 18S rRNA (Henras et al. 2015; Ameismeier et al. 2020). Consistent with this conserved paradigm, ZmNOB1 localized predominantly to the cytoplasm and physically interacted with the conserved assembly factor PNO1, indicating that maize NOB1 operates within cytoplasmic pre-40S maturation complexes (Figs. 2 and 6). Structural studies in human cells demonstrated that PNO1 masks the D-site and prevents premature cleavage by NOB1 until conformational remodeling activates the pre-40S particle (Ameismeier et al. 2018, 2020). Our interaction analyses suggest that a similar regulatory mechanism is likely conserved in maize. Moreover, the strong enrichment of 18S-containing pre-rRNA intermediates in ZmNOB1 immunoprecipitates further supports a direct role for ZmNOB1 in late pre-rRNA maturation.

Importantly, simultaneous impairment of ZmNOB1 activity resulted in widespread disruption of pre-rRNA processing, including defective D-site cleavage and substantial alterations in both upstream and downstream processing intermediates (Fig. 5). The accumulation of aberrant 18S-A2, P′-A3, and 27S-related precursors indicates that late-stage maturation defects exert an indirectly effect on early pre-rRNA processing events, thereby perturbing overall ribosome biogenesis dynamics. Although distinct stages of ribosome assembly are spatially and temporally compartmentalized, several mechanisms could account for such inter-stage feedback regulation. First, many assembly factors shuttle dynamically between the nucleus and cytoplasm (Senger et al. 2001; Kallstrom et al. 2003). Blocking late cytoplasmic maturation steps may trap these factors on aberrant pre-ribosomal particles in the cytoplasm, preventing their nuclear recycling and depletion of the nuclear pool required for early-to-mid processing events. Second, pre-ribosomal nuclear export is an active, regulated process rather than passive diffusion. Cytoplasmic maturation defects can impair nuclear export efficiency via retrograde signaling, leading to the nuclear retention and accumulation of unprocessed precursors (Peña et al. 2017). Finally, disrupted ribosome assembly can trigger nucleolar stress responses, transmitting signaling cascades from the cytoplasm back to the nucleolus to downregulate early rRNA processing steps (Zakrzewska-Placzek et al. 2025). Similar coupling between ribosome assembly checkpoints and global processing homeostasis has been described in other eukaryotic systems (Henras et al. 2015). Our findings therefore suggest that ZmNOB1 functions not merely as a terminal endonuclease, but rather as an integral component of a surveillance network coordinating late 40S maturation with overall ribosome assembly fidelity. Interestingly, although defective D-site cleavage was readily detectable in mutant kernels, aberrant intermediates accumulated at relatively low abundance compared with mature rRNA species. These observations suggest that the severe developmental defects in *Aabb* kernels are unlikely to arise solely from impaired 18S maturation itself, but instead reflect broader defects in ribosome assembly and translational competence resulting from compromised late-stage ribosome quality control.

### The potential connection between ZmNOB1 and translation

In eukaryotes, the mature 40S ribosomal subunit associates with initiation factors (eIF1, eIF1A, eIF3, and eIF5) together with the ternary complex (eIF2·GTP·Met-tRNA_i_^Met^) to assemble the 43S pre-initiation complex (Aitken and Lorsch 2012; Wang et al. 2022b). Following recruitment to the mRNA, the 43S complex scans the transcript to identify the translation start codon, thereby forming the 48S initiation complex and promoting subsequent joining of the 60S LSU to generate a translationally competent 80S ribosome (Wang et al. 2022b). Polysome profiling revealed pronounced accumulation of free 40S and 60S ribosomal subunits accompanied by a substantial reduction in 80S monosomes in both *Aabb* and *aaBb* mutants (Fig. 7). These observations suggest that compromised NOB1 activity impairs either subunit joining or efficient recruitment of the 40S subunit to mRNA during translation initiation. Alternatively, defective maturation of the 40S subunit itself may diminish its capacity to properly engage the mRNA 5′ end, thereby limiting productive 80S assembly (Fig. 8). The abnormal accumulation of free ribosomal subunits may additionally trigger feedback inhibition of pre-rRNA processing pathways, potentially accounting for the widespread defects observed across multiple cleavage sites.

**Figure 8:**
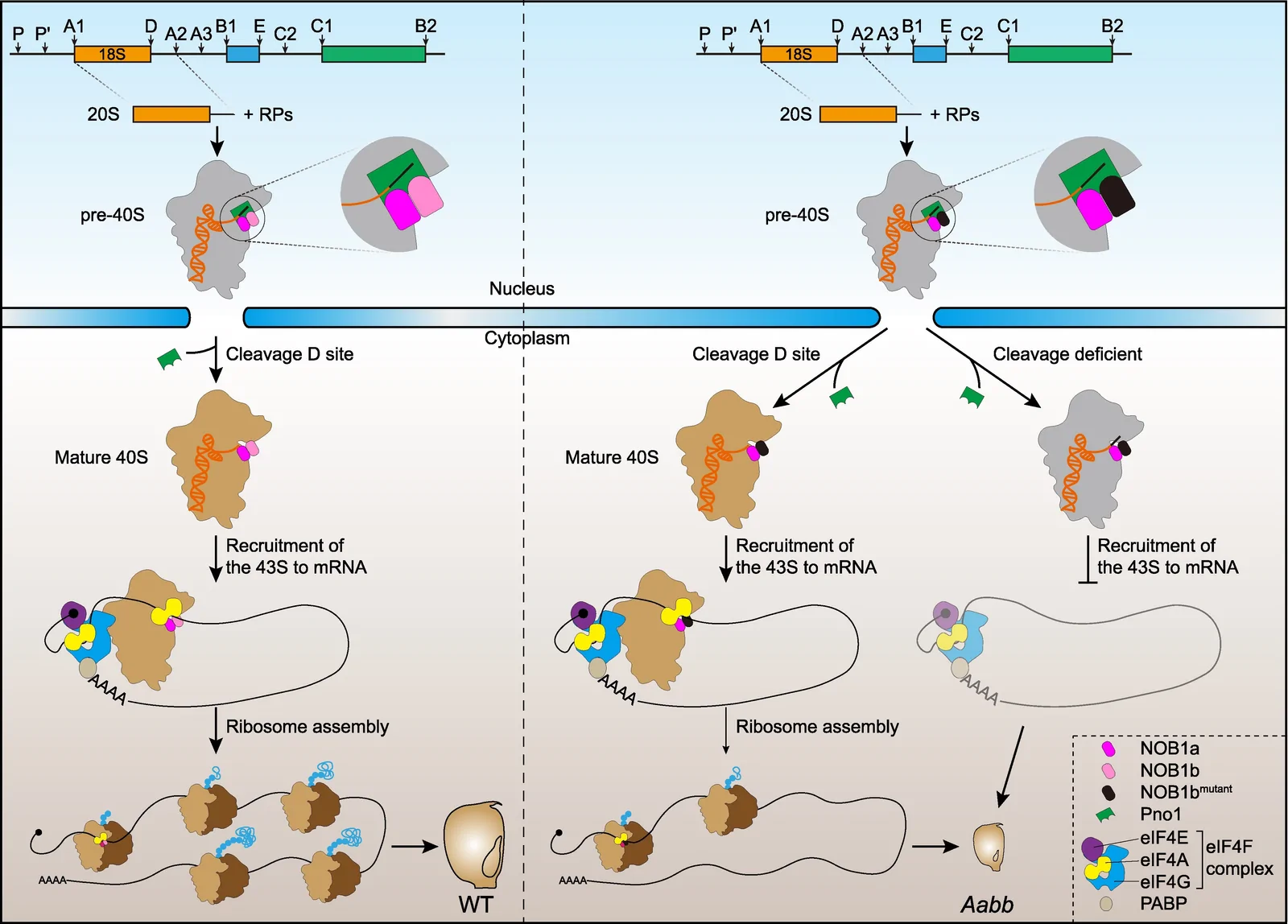
Proposed model for NOB1-mediated regulation of ribosome maturation and kernel development in maize. 45S pre-rRNA is transcribed in the nucleus and processed into 20S pre-rRNA, which associates with ribosomal proteins to form the pre-40S ribosomal particle. Pre-40S and pre-60S particles are subsequently exported to the cytoplasm for final maturation. In the cytoplasm, NOB1 associated with the pre-40S particle is initially inhibited by PNO1. Following remodeling by additional ribosome assembly factors, PNO1 dissociates, allowing NOB1 to cleave the 3′ extension of 20S pre-rRNA to generate mature 18S rRNA. The mature 40S ribosomal subunit is then recruited to the 5′ end of mRNA through interactions with the eIF4F complex and eIF4A at the mRNA entry channel. The 40S subunit scans the mRNA in the 5′-to-3′ direction before recruitment of the mature 60S subunit to assemble the translationally competent 80S ribosome.

Recent proximity-proteomics analyses in human cells identified numerous translation-associated proteins, including eIF4G2, eIF4B, eIF4G3, EIF5, and eIF3L, within NOB1- and PNO1-associated complexes (Xu et al. 2025), suggesting an unexpected functional connection between late-stage ribosome maturation and translation initiation. In eukaryotes, the eIF4F complex, composed of eIF4E, eIF4G, and eIF4A, recognizes the mRNA 5′ cap structure and facilitates unwinding of local secondary structures to promote ribosome loading (Marintchev et al. 2009; Brito Querido et al. 2020). Among these components, the DEAD-box RNA helicase eIF4A plays a pivotal role by stimulating recruitment of the 43S pre-initiation complex near the m^7^G cap and enabling directional scanning along the mRNA (García-García et al. 2015; Yourik et al. 2017). Our findings indicate that NOB1 and eIF4A co-localize to the cytoplasm and co-fractionate with 40S ribosomal subunits (Fig. 7a; Figure S14). Interaction assays demonstrated that ZmNOB1 directly associates with eIF4A and is further linked to additional components of the eIF4F complex, raising the possibility that NOB1 contributes to recruitment or activation of translation-competent 43S complexes in maize. Intriguingly, recent structural analyses of the human 48S initiation complex identified a second eIF4A molecule positioned downstream of the mRNA entry channel, implying that eIF4A may function beyond its canonical role within eIF4F (Brito Querido et al. 2024b). Whether the NOB1–eIF4A interaction contributes to ribosome loading, scanning efficiency, or quality control of newly matured 40S subunits remains an important question for future investigation.

### Defective ribosome maturation constrains translational capacity during kernel filling

Sustained ribosome production is essential for maintaining kernel filling and biomass accumulation (Wang et al. 2018; Liu et al. 2022; Wang et al. 2022a; Wang et al. 2024). Our results demonstrate that impaired ZmNOB1 function severely compromises this process. Consistent with the polysome profiling results, puromycin incorporation assays revealed a global reduction in protein synthesis, together with a significant decrease in the levels of mRNAs associated with monosomes and polysomes (Fig. 7). These observations establish that defects in pre-rRNA maturation directly constrain translational capacity during kernel development. The developmental consequences of this translational impairment were particularly evident in endosperm metabolism. *Aabb* kernels exhibited severe reductions in starch accumulation, abnormal starch granule morphology, disrupted protein body formation, and decreased abundance of key biosynthetic enzymes, including SH2, BT2, SBE1, and SS1 (Fig. 7). Although ZmNOB1, ZmRcl1 (Wang et al. 2022a), and ZmDek51 (Wang et al. 2024) differ in subcellular localization and in the combination of processing sites they directly act upon, mutations in these genes converge on remarkably similar downstream consequences. Notably, transcript abundance did not consistently correlate with protein accumulation (Schwanhäusser et al. 2011; Zhang et al. 2025), indicating that translational repression, rather than transcriptional alteration, represents the primary driver of the mutant phenotype. Through its interaction with eIF4A, NOB1 may enhance the translational efficiency of either global or specific subsets of mRNAs. Under conditions of reduced NOB1 activity, even partially matured 40S subunits may fail to efficiently engage the translation initiation machinery, resulting in a global decline in translational capacity.

Collectively, our findings support a model in which maize NOB1 proteins constitute a multilayered regulatory module integrating ribosome biogenesis with translational control (Fig. 8). Following gene duplication, ZmNOB1a and ZmNOB1b retained their conserved indispensable function in late pre-40S maturation while undergoing partial functional divergence that contributes to developmental robustness and phenotypic specialization. More importantly, the physical association between NOB1 and components of the translation initiation machinery establishes a direct mechanistic link between ribosome maturation and translational competence. These findings expand current understanding of how ribosome biogenesis is coordinated with translation initiation in crops and further establish NOB1 as a central regulatory nexus linking late pre-40S maturation with translational competence.

## Materials and methods

### Plant materials and growth conditions

Four EMS-induced nonsense alleles of *zmnob1a* (*aa-1* and *aa-2*) and *zmnob1b* (*bb-1* and *bb-2*) were isolated from the B73 background. Double-heterozygous plants were generated by crossing the single mutants. Transgenic maize overexpressing *proUbi::3×Flag-ZmNOB1-GFP* was generated via *Agrobacterium*-mediated transformation. Maize plants were grown under field conditions in Hefei and Sanya, China. *N. benthamiana* plants were cultivated in a greenhouse at 25 °C under a 16-h-light/8-h-dark photoperiod.

### Phylogenetic analysis and sequence alignment

NOB1 homologs were retrieved from the UniProt database. Phylogenetic analysis was conducted using MEGA5 with the neighbor-joining method and 1,000 bootstrap replicates. Multiple sequence alignments were generated with ClustalW and visualized with ESPript 3.2. Conserved domains were predicted using SMART, and protein structures were modeled using AlphaFold and visualized in PyMOL.

### RNA EMSA and endonuclease assays

The coding sequences of *ZmNOB1a* and *ZmNOB1b* were cloned into the pMAL-c2X vector and expressed in *E. coli* Rosetta (DE3) cells (WEIDI, EC1010). Protein expression was induced with 0.1 mM IPTG at 16 °C overnight. Recombinant MBP fusion proteins were purified using amylose resin (New England Biolabs, USA, E8021) according to the manufacturer's instructions. RNA probes corresponding to the maize pre-rRNA sequences flanking the D-site were synthesized with biotin or Cy3 labels. RNA EMSA was performed according to an established protocol with minor modifications (Liu et al. 2025). For each EMSA reaction, 0.5 μM of labeled RNA probe was incubated with increasing amounts (0.1 and 0.5 μM) of purified MBP, ZmNOB1a-MBP, or ZmNOB1b-MBP proteins in binding buffer (10 mM HEPES, pH 8.0, 50 mM KCl, 1 mM EDTA, 0.05% Triton X-100, 10 μg/mL salmon sperm DNA, 5% glycerol, 1 mM DTT, 1 × protease inhibitor cocktail, and 40 U/mL RNasin). For competition assays, a 50-fold molar excess of unlabeled homologous RNA probe was added together with the biotin-labeled probe to the reaction mixture. All reactions were incubated on ice for 1 h. The samples were resolved on 6% native polyacrylamide gels and transferred to Hybond-N^+^ membranes (GE Healthcare, RPN303B). Signals were detected using a Chemiluminescent Nucleic Acid Detection Kit (Thermo Scientific, USA, 89880) following the manufacturer's instructions.

The endonuclease activity assay was performed according to an established protocol with minor modifications (Pertschy et al. 2009). Briefly, labeled RNA probes were incubated with recombinant proteins in reaction buffer (25 mM Tris-HCl, pH 7.6, 75 mM NaCl, 2 mM DTT, 100 μg/mL BSA, 40 U/mL RNasin, 5% glycerol, 0.05% NP-40, 0.3 μM tRNA). Unless otherwise indicated, the reactions were supplemented with 5 mM MnCl_2_. After incubation at 37 °C for 1 h, the cleavage products were resolved by 10% denaturing urea-PAGE and visualized by ethidium bromide staining or fluorescence scanning using a Typhoon imaging system (GE Healthcare).

### RIP-qPCR

RIP-qPCR assays were performed essentially as described previously (Mermaz et al. 2018) with minor modifications. Approximately 2 g of fresh 16-DAP kernels expressing *proUbi::3×Flag-ZmNOB1b-GFP* were ground in liquid nitrogen and homogenized in lysis buffer (20 mM Tris-HCl, pH 7.5, 150 mM NaCl, 2.5 mM MgCl_2_, 0.5% Triton X-100, 10% glycerol, 0.5 mM DTT, 1 mM PMSF, 1 × protease inhibitor cocktail, and 40 U/mL RNasin). After centrifugation, the supernatant was incubated with anti-GFP antibody-conjugated Dynabeads Protein G (Thermo Scientific, USA, 10004D) at 4 °C for 2 h, with nonspecific IgG used as a negative control. Following extensive washing, the immunoprecipitated RNA was purified and analyzed by RT-qPCR using primers spanning distinct regions of the 45S pre-rRNA.

### Protein–protein interaction

For Y2H assays, the full-length or truncated coding sequences were cloned into the pGBKT7 and pGADT7 vectors and co-transformed into the Y2H Gold yeast strain using the PEG/LiAc method. Transformants were selected on double-dropout (DDO) medium and further assayed on quadruple-dropout (QDO) medium after incubation at 30 °C for 3 to 5 d.

LCI assays were performed as previously described (Chen et al. 2008). The coding sequences were cloned into the pCAMBIA-nLuc or pCAMBIA-cLuc vectors and transiently co-expressed in *N. benthamiana* leaves via *Agrobacterium tumefaciens* GV3101 infiltration. Luminescence signals were detected 48 h after infiltration using a low-light CCD imaging system (Tanon 5200, Shanghai, China). For BiFC assays, coding sequences without stop codons were fused to split-YFP vectors and transiently expressed in tobacco leaves. YFP fluorescence was observed 48 h after infiltration using confocal microscopy (LSM880, Zeiss, Germany). Co-IP assays were performed using tobacco leaves transiently co-expressing GFP- and Myc-tagged proteins. Total proteins were extracted and incubated with anti-Myc magnetic beads (Bimake, Shanghai, China, B26301) at 4 °C for 2 h. After washing, the immunoprecipitated proteins were analyzed by SDS-PAGE and immunoblotting.

### Nuclear and cytosolic fractionation

The nuclear and cytosolic fractionation was performed based on a previously established protocol (Zhao et al. 2018).

### Light microscopy of cytological sections

Paraffin sectioning of maize kernels was performed as follows. Samples were fixed in formalin-acetic-alcohol solution (5 mL 37% formaldehyde, 5 mL glacial acetic acid, and 90 mL 70% ethanol), subjected to vacuum infiltration, and then stored at 4 °C. The fixed materials were subsequently dehydrated through a graded ethanol series, cleared with xylene, infiltrated with paraffin, and embedded. Serial sections were cut using a paraffin microtome, stained with toluidine blue, and observed under a light microscope (MOTIC, SMZ-171).

Transmission electron microscopy and scanning electron microscopy observations were performed according to a previously established protocol (Wang et al. 2022a).

### Protein and starch measurement

Zein and non-zein proteins were extracted from mature kernels as previously described (Wang et al. 2022a). Protein concentrations were measured using the BCA Protein Assay Kit (Sangon, Shanghai, China, C503051) according to the manufacturer's instructions.

Total starch (Sangon, Shanghai, China, D799325) and amylose (Sangon, Shanghai, China, D799146) contents in mature kernels were determined according to the manufacturer's manual.

### Pollen vitality analysis

Mature maize pollen grains were collected on a clean microscope slide. One or two drops of I_2_-KI solution were applied directly onto the sample and allowed to react for approximately 2 min. A cover slip was then gently placed over the stained pollen. The slide was observed under a compound light microscope. Starch granules stored within viable pollen grains stained a distinct blue-black or deep purple color, whereas the pollen walls remained unstained.

### dCAPS

A PCR primer containing an intentional single-base mismatch was designed using dCAPS Finder 2.0. This mismatch, together with the target SNP, either creates or eliminates a restriction enzyme recognition site depending on the allele present. The target DNA region was then amplified by standard PCR. Subsequently, the PCR products were digested with the corresponding restriction enzyme, and the resulting fragments were separated by agarose gel electrophoresis.

### Subcellular localization

The coding sequences of *ZmNOB1a* and *ZmNOB1b* were subcloned into the pCAMBIA1300-GFP vector. The correctly constructed plasmids were then transformed into *A. tumefaciens* GV3101 competent cells. Subsequently, the bacterial suspension was infiltrated into the lower epidermal cells of 4-wk-old tobacco leaves. After 48 h, observations and images were captured using an LSM880 confocal microscope (Zeiss, Jena, Germany). Following a previously described method (Yoo et al. 2007), protoplasts were isolated from B73 maize etiolated seedlings. The plasmids ZmNOB1-GFP and RRP7-RFP were co-transfected into the protoplasts using PEG-mediated transformation. After 16 h of incubation in the dark, the samples were observed and photographed using an LSM880 confocal microscope (Zeiss, Jena, Germany). At least, 6 maize protoplasts were observed with similar results.

### RNA extraction, RT-qPCR, circular RT-PCR, and northern blot

Total RNA was extracted from developing maize kernels using a commercial RNA extraction kit (Vazyme, Nanjing, China, RC401-01) according to the manufacturer's instructions. For RT-qPCR, 1 μg of total RNA was reverse-transcribed into cDNA using HiScript III RT SuperMix (Vazyme, Nanjing, China, R323-01), and amplification was performed using SYBR Green Master Mix on a Roche LightCycler 480 system (Roche, Basel, Switzerland). Relative transcript levels were normalized against *Ubi*. For each experiment, 3 biological replicates from independent ears were analyzed.

Circular RT-PCR was performed to characterize pre-rRNA processing intermediates. Briefly, total RNA was circularized using T4 RNA ligase I, reverse-transcribed with gene-specific primers, and amplified using PrimeSTAR GXL polymerase (Takara, Japan, R050A). PCR products were purified, cloned, and sequenced to identify rRNA processing sites.

For northern blot analysis, total RNA was separated on formaldehyde-agarose gels and transferred to Hybond-N^+^ membranes (GE Healthcare, RPN303B). The membranes were hybridized with digoxigenin-labeled probes corresponding to distinct regions of pre-rRNA intermediates. Signals were detected using anti-DIG alkaline phosphatase-conjugated antibodies (Roche, Germany, 11093274910) and chemiluminescence substrates (Roche, Germany, 12041677001).

### Polysome profiling

Polysome profiles were analyzed according to a described protocol (Wang et al. 2022a). 2 g of 16-DAP kernels from B73 and mutants (*Aabb* and *aaBb*) were used for polysome isolation.

For protein distribution analysis, a total of 15 fractions (750 μL each) were collected from the polysome gradient using a fraction collector. Proteins from individual fractions were precipitated with isopropanol and subsequently analyzed by Western blotting.

To extract RNA from gradient fractions corresponding to monosome and polysome pools, 1 mL of RNAiso Plus reagent (Takara, Japan, 9108) was added to each collected fraction. The mixture was vigorously vortexed and incubated at room temperature for 5 min to ensure complete dissociation of nucleoprotein complexes. Chloroform was then added, and the samples were shaken vigorously for 15 s, followed by incubation at room temperature for 2 to 3 min. After centrifugation at 12,000 × *g* for 15 min at 4 °C, the aqueous phase was carefully transferred to a fresh tube. RNA was precipitated by adding isopropanol and incubating at room temperature for 10 min, followed by centrifugation at 12,000 × *g* for 10 min at 4 °C. The RNA pellet was washed with 75% ethanol, air-dried, and dissolved in RNase-free water. RNA concentration and purity were determined using a NanoDrop spectrophotometer. The extracted RNA was then subjected to RT-qPCR analysis.

### Puromycin labeling assay

7-d-old B73 and mutant seedlings grown on germination paper were placed in centrifuge tubes, and 1/2 MS liquid medium was added to cover the roots. The seedlings were incubated for 15 min, and then transferred to fresh 1/2 MS liquid medium containing 1 μg/mL puromycin (Solarbio, Beijing, China, P8230) for translational inhibition. The samples were subjected to 3 additional rounds of vacuum infiltration: each round consisted of 1 min of vacuum followed by 1 min of decompression at atmospheric pressure. Samples were collected 30 min after the third vacuum infiltration, immediately frozen in liquid nitrogen, and then processed for protein extraction and protein immunoblotting. Equal amounts of total protein extracted from each sample were resolved by SDS-PAGE, electro-transferred to a PVDF membrane, and immunoblotted with an anti-puromycin antibody (Merck, USA, ZMS1016).

### Immunoblot analysis

Total proteins from maize kernels or tobacco leaves were extracted using extraction buffer (50 mM Tris-HCl, pH 8.8, 10% glycerol, 4% SDS, and β-mercaptoethanol). Protein samples were separated by SDS-PAGE and transferred onto PVDF membranes (Millipore, Billerica, MA, USA, IPVH00010). The following antibodies were used: anti-Actin (CWBIO, CW0264M), anti-Histone H3 (HUABIO, ET1701-64), anti-GFP (TransGen, HT801-1), anti-Myc (HUABIO, R1208-1), anti-eIF4A (ABclonal, A5294), anti-RPS6 (ABclonal, A11874), anti-RPL13 (Agrisera, AS13 2650), anti-NOB1 (ABclonal, A17690), anti-PUM24 (Orizymes, 240949), anti-O2 (ABclonal, WG-00673D), and antibodies against proteins involved in starch biosynthesis (Zhang et al. 2016). Signals were detected with High-sig ECL Western Blotting Substrate (Tanon, Shanghai, China, 180-5001) and visualized using a Tanon 5200 chemiluminescent imaging system (Tanon, Shanghai, China).

### Statistical analysis

All statistical analyses were conducted using GraphPad Prism version 8. Two-tailed Student's *t*-test or one-way ANOVA were performed as appropriate. The detailed statistical results are shown in Data Set S5. ImageJ software was used to measure the peak area of the ribosome profile.

### Accession numbers

Sequence data from this article can be searched in the MaizeGDB database under the following accession numbers: *ZmNOB1a* (*Zm00001d014988*); *ZmNOB1b* (*Zm00001d054047*); *Ubi* (*Zm00001d015327*); *ZmRio2* (*Zm00001eb145320*); *ZmPno1-1* (*Zm00001eb369690*); *ZmPno1-2* (*Zm00001eb151110*); *ZmEnp1* (*Zm00001eb065940*); *ZmRps5* (*Zm00001eb334050*); *ZmO2* (*Zm00001d018971*); *ZmSH1* (*Zm00001d045042*); *ZmSH2* (*Zm00001d044129*); *ZmBT2* (*Zm00001d050032*); *ZmSBE1* (*Zm00001d014844*); *ZmSBE2* (*Zm00001d016684*); *ZmSS1* (*Zm00001d045261*); *ZmSS2* (*Zm00001d002256*); *ZmSS3* (*Zm00001d026337*); *ZmPPDK1* (*Zm00001d038163*); *ZmPPDK2* (*Zm00001d010321*); *ZmSU1* (*Zm00001d049753*); *ZmGBSS1* (*Zm00001d045462*); *ZmPGD1* (*Zm00001d035925*); *ZmPGD2* (*Zm00001d042184*); *ZmPGD3* (*Zm00001d049187*); *ZmOHP1* (*Zm00001d034457*); *ZmOHP2* (*Zm00001d013074*); *ZmPBF* (*Zm00001d005100*); *ZmeIF4A*-1 (*Zm00001eb232200*); *ZmeIF4A*-2 (*Zm00001eb272920*); *ZmeIF4A*-3 (*Zm00001eb226920*).

## Supplementary Material

koag330_Supplementary_Data

## Data Availability

The data underlying this article are available in the article and in its online supplementary material. The data underlying this article will be shared on reasonable request to the corresponding author J.Y.
