# Supplementary material for: Maize endonuclease NOB1 couples ribosome biogenesis with translational regulation during kernel development
Source: Plant Cell. 2026 Oct 28;38(11):koag330. doi: 10.1093/plcell/koag330 (PMC13760097; doi:10.1093/plcell/koag330)
Supplement: Supplementary file 1 [file koag330_supplementary_data.zip › Supplemental Figure(s).doc]

**Supplemental Figure S1. Identification and structural characterization of maize NOB1 homologs. (Supports Figure 1 and Figure 2)**

**(A)** Schematic representation of the domain organization of the two maize NOB1 paralogs. PIN, PilT N-terminus endonuclease domain; Zn-bind, Zn-ribbon domain. Conserved domains were predicted using SMART (<https://smart.embl.de/>). **(B)** Multiple sequence alignment of ZmNOB1a, ZmNOB1b, and their homologs from *Arabidopsis thaliana* and *Saccharomyces cerevisiae* generated using ClustalW. Conserved residues within the PIN and Zn-ribbon domains are highlighted. **(C)** Phylogenetic analysis of NOB1 homologs from angiosperms, bryophytes, chlorophytes, mammals, archaea, fungi, and nematodes. The phylogenetic tree was generated in MEGA-X using the maximum-likelihood method with 1000 bootstrap replicates. Bootstrap values are indicated at the nodes. Protein sequences used for the analysis are listed in Supplemental Data Set 2. **(D)** The PIN domain structures of ZmNOB1a (56-143 aa), ZmNOB1b (70-157 aa), AtNOB1 (47-134 aa), and ScNOB1 (12-102 aa) were obtained from AlphaFold (<https://www.alphafold.ebi.ac.uk/>), aligned and visualized using PyMOL.

**Supplemental Figure S2.** **ZmNOB1 specifically recognizes maize pre-rRNA substrates.** **(Supports Figure 1)**

**(A)** Affinity purification of recombinant ZmNOB1-MBP fusion proteins expressed in *E. coli*. Proteins were separated by SDS-PAGE and visualized by Coomassie Brilliant Blue staining. Lane 1, uninduced cell lysate; Lane 2, induced cell lysate; Lane 3, purified fusion protein eluted from amylose resin with maltose. Black triangles indicate the target proteins. **(B and C)** Sequences and schematic structures of the 5′ biotin-labeled RNA probes used for RNA-binding and cleavage assays. Probes were derived from maize (B) or yeast (C) sequences spanning the 3′ end of mature 18S rRNA (bold) and the 5′ region of ITS1 (regular font). An artificial stem-loop structure was incorporated into each substrate to stabilize the D-site-containing fragment. **(D)** EMSA assays using identical protein concentration gradients incubated with maize-derived (left) or yeast-derived (right) RNA probes. **(E)** Sequence comparison of the 3′ end of 18S rRNA and ITS1 regions between maize and yeast.

**Supplemental Figure S3. *In vitro* validation of ZmNOB1 endonuclease activity.** **(Supports Figure 1)**

**(A)** Sequence of the Cy3-labeled RNA substrate used for endonuclease assays. The probe contains maize sequences spanning the 3′ end of mature 18S rRNA (bold) and the 5′ region of ITS1 (regular font). **(B)** *In vitro* cleavage assays performed by incubating the RNA substrate with excess ZmNOB1a-MBP or ZmNOB1b-MBP protein to assess endonuclease activity.

**Supplemental Figure S4. Subcellular localization of ZmNOB1a and ZmNOB1b in *N. benthamiana* leaf epidermal cells.** **(Supports Figure 2)**

ZmNOB1-GFP fusion proteins were co-expressed with Arabidopsis RRP7 protein. Chlorophyll autofluorescence is shown in blue. Scale bar = 50 μm.

**Supplemental Figure S5.** **Comparison of kernel phenotypes between B73 and *zmnob1a/b* mutants. (Supports Figure 3)**

**(A-C)** Kernel length (A), width (B), and thickness (C) in the B73 and *zmnob1a/b* mutants. Scale bar = 1 cm. **(D-F)** Quantitative measurements of kernel length (D), width (E), and thickness (F) in the B73 and *zmnob1a/b* mutants. Values represent means ± SD (n = 10). Statistical significance was determined by one-way ANOVA relative to B73 (ns, not significant; **P* < 0.05; ****P* < 0.001; *****P* < 0.0001).

**Supplemental Figure S6. Cytological characterization of *zmnob1* mutant kernels.** **(Supports Figure 3)**

**(A)** Longitudinal hand-dissected sections of mature kernels from B73 and *zmnob1* mutants. Embryo regions are outlined by magenta dashed lines. Samples were stained with 2,3,5-triphenyltetrazolium chloride (TTC); red staining indicates viable embryo tissues with intact membrane permeability. Scale bar = 2 mm. **(B)** Longitudinal paraffin sections of B73 and *zmnob1* kernels at 19 DAP. Enlarged embryo images highlight tissue organization and developmental structures. SC, scutellum; Co, coleoptile; Lp, leaf primordia; SAM, shoot apical meristem; Rp, root primordium. Scale bar = 0.5 mm.

**Supplemental Figure S7. Mapping of pre-rRNA processing intermediates in *zmnob1* mutants by circular RT-PCR.** (**Supports Figure 3 and Figure 5)**

Mapping of the 5′ and 3′ termini of pre-rRNA intermediates in B73, *aa-1*, *aa-2*, *bb-1*, and *bb-2* kernels by circular RT-PCR. Diagrams on the left indicate the primer combinations used for reverse transcription of circularized RNA and subsequent amplification of specific pre-rRNA intermediates.

**Supplemental Figure S8. Sequencing analysis of 18S rRNA termini in *zmnob1* mutants. (Supports Figure 3)**

PCR-amplified fragments corresponding to 18S rRNA termini were cloned into a blunt-end vector and subjected to Sanger sequencing. Magenta nucleotides indicate aberrant cleavage sites relative to the reference 18S rRNA sequence. Numbers outside parentheses indicate the total number of sequenced clones, whereas magenta numbers within parentheses indicate clones exhibiting abnormal D-site cleavage.

**Supplemental Figure S9. The *Aabb* mutation disrupts kernel and plant development. (Supports Figure 4)**

**(A–C)** Genotypic identification of mutant kernels by dCAPS analysis and sequencing. **(D–F)** Representative mature kernels showing differences in kernel length (D), width (E), and thickness (F) between B73 and *Aabb*. Scale bar = 1 cm. **(G–I)** Quantification of kernel length (G), width (H), and thickness (I) in B73 and *Aabb* kernels. Values represent means ± SD (n = 10). Statistical significance was determined using two-tailed Student’s *t*-test (ns, not significant; ***P* < 0.01; *****P* < 0.0001). **(J)** Comparison of plant architecture between B73 and *Aabb* plants. Scale bar = 20 cm. **(K)** Comparison of tassel morphology between B73 and *Aabb* plants. Scale bar = 5 cm.

**Supplemental Figure S10. Phenotypic characterization of genetic crosses involving *AaBb* and *zmnob1* single mutants.** **(Supports Figure 4)**

**(A)** Mature ears derived from genetic crosses, with abnormal kernels indicated by white arrows. Scale bar = 1 cm. **(B)** Mature ears from self-pollinated *aa1Bb1* plants. Abnormal kernels are indicated by white arrows. Scale bar = 1 cm. **(C–E)** Representative kernels showing differences in kernel length (C), width (D), and thickness (E) between B73 and *aaBb*. Scale bar = 1 cm. **(F–H)** Quantitative analysis of kernel length (F), width (G), and thickness (H) in B73 and *aaBb* kernels. Values represent means ± SD (n = 10). Statistical significance was determined using two-tailed Student’s *t*-test (ns, not significant; **P* < 0.05; ****P* < 0.001). **(I)** Comparison of hundred-kernel weight between B73 and *aaBb* mature kernels. Data are presented as means ± SD (n = 10). Statistical significance was determined using two-tailed Student’s *t*-test (*****P* < 0.0001). **(J)** Phenotypic analysis of crosses using *AaBb* as the male parent and *zmnob1* single mutants as the female parent. Scale bar = 1 cm.

**Supplemental Figure S11. Defective D-site cleavage in *zmnob1* mutant kernels. (Supports Figure 5)**

Electrophoretic analysis of PCR products amplified with primers 18SL0/R8 from individual cloned fragments. PCR products generated with primers CR2/R8 were ligated into a cloning vector prior to analysis. Correct D-site cleavage yields a 155-bp fragment.

**Supplemental Figure S12. RT-qPCR analysis of the RBFs and RPs gene expression in B73, *Aabb*, and *aaBb* 19-DAP kernels. (Supports Figure 7)**

Expression levels were normalized to *Ubi*. Data represent means ± SD (n = 3 biological replicates). Statistical significance was determined by two-tailed Student’s *t*-test (ns, not significant; **P* < 0.05; ***P* < 0.01; ****P* < 0.001; *****P* < 0.0001).

**Supplemental Figure S13. Expression levels of zein genes and their regulatory factors as well as starch synthesis-related genes in B73, *Aabb*, and *aaBb* 19-DAP kernels. (Supports Figure 7)**

Expression levels were normalized to *Ubi*. Data represent means ± SD (n = 3 biological replicates). Statistical significance was determined by two-tailed Student’s *t*-test (ns, not significant; **P* < 0.05; ***P* < 0.01; ****P* < 0.001; *****P* < 0.0001).

**Supplemental Figure S14. Co-localization of ZmeIF4As and ZmNOB1a in maize protoplasts. (Supports Figure 7)**

At least five protoplasts were observed with similar results. Scale bar = 10 μm.

**Supplemental Figure S15. Interactions between ZmNOB1 and the eIF4F complex examined by Y2H and LCI assays.** **(Supports Figure 7)**

**(A)** Y2H assay of the interaction between ZmNOB1 and the eIF4F complex. DDO, double dropout; QDO, quadruple dropout. **(B)** LCI assay of the interaction between ZmNOB1 and the eIF4F complex in *N. benthamiana*.

**Table S1. Transmission of the *ab* haplotype through male and female gametophytes.** **(Supports Figure 4)**

**(A)** Progeny from reciprocal crosses with B73 as the female parent and *AaBb* as the male parent. **(B)** Progeny from crosses with *AaBb* as the female parent and B73 as the male parent. Chi-square (*χ²*) and *P* values are shown for each individual cross, testing the expected 1:1:1:1 Mendelian segregation ratio. Total counts and combined chi-square statistics are provided at the bottom. Data represent four independent combinations of mutant alleles for each gene.
